# Rapid Downregulation of H3K4me3 Binding to Immunoregulatory Genes in Altered Gravity in Primary Human M1 Macrophages

**DOI:** 10.3390/ijms24010603

**Published:** 2022-12-29

**Authors:** Christian Vahlensieck, Cora Sandra Thiel, Swantje Christoffel, Sabrina Herbst, Jennifer Polzer, Beatrice Astrid Lauber, Saskia Wolter, Liliana Elisabeth Layer, Jochen Hinkelbein, Svantje Tauber, Oliver Ullrich

**Affiliations:** 1 Institute of Anatomy, Faculty of Medicine, University of Zurich, Winterthurerstrasse 190, 8057 Zurich, Switzerland; 2 Department of Machine Design, Engineering Design and Product Development, Institute of Mechanical Engineering, Otto-von-Guericke-University Magdeburg, Universitätsplatz 2, 39106 Magdeburg, Germany; 3 Space Life Sciences Laboratory (SLSL), Kennedy Space Center, 505 Odyssey Way, Exploration Park, Merritt Island, FL 32953, USA; 4 UZH Space Hub, Air Force Center, Air Base Dübendorf, Überlandstrasse 270, 8600 Dubendorf, Switzerland; 5 Ernst-Abbe-Hochschule (EAH) Jena, Department of Industrial Engineering, Carl-Zeiss-Promenade 2, 07745 Jena, Germany; 6 Department for Anaesthesiology and Intensive Care Medicine, University Hospital of Cologne, 50937 Cologne, Germany; 7 Zurich Center for Integrative Human Physiology (ZIHP), University of Zurich, Winterthurerstrasse 190, 8057 Zurich, Switzerland

**Keywords:** microgravity, immune system, epigenetics, parabolic flight, ChIP-seq, formaldehyde fixation

## Abstract

The sensitivity of human immune system cells to gravity changes has been investigated in numerous studies. Human macrophages mediate innate and thus rapid immune defense on the one hand and activate T- and B-cell-based adaptive immune response on the other hand. In this process they finally act as immunoeffector cells, and are essential for tissue regeneration and remodeling. Recently, we demonstrated in the human Jurkat T cell line that genes are differentially regulated in cluster structures under altered gravity. In order to study an in vivo near system of immunologically relevant human cells under physically real microgravity, we performed parabolic flight experiments with primary human M1 macrophages under highly standardized conditions and performed chromatin immunoprecipitation DNA sequencing (ChIP-Seq) for whole-genome epigenetic detection of the DNA-binding loci of the main transcription complex RNA polymerase II and the transcription-associated epigenetic chromatin modification H3K4me3. We identified an overall downregulation of H3K4me3 binding loci in altered gravity, which were unequally distributed inter- and intrachromosomally throughout the genome. Three-quarters of all affected loci were located on the p arm of the chromosomes chr5, chr6, chr9, and chr19. The genomic distribution of the downregulated H3K4me3 loci corresponds to a substantial extent to immunoregulatory genes. In microgravity, analysis of RNA polymerase II binding showed increased binding to multiple loci at coding sequences but decreased binding to central noncoding regions. Detection of altered DNA binding of RNA polymerase II provided direct evidence that gravity changes can lead to altered transcription. Based on this study, we hypothesize that the rapid transcriptional response to changing gravitational forces is specifically encoded in the epigenetic organization of chromatin.

## 1. Introduction

The immune system is one of the systems critically affected under spaceflight conditions [1,2,3], and the sensitivity of human immune system cells to gravity changes and microgravity has been confirmed in numerous studies in different immune cell systems, research platforms, and in terms of many cellular and molecular functions [3,4]. The primary human M1 macrophages investigated in this study mediate innate and thus rapid and immediate immune defense on the one hand, and activate T- and B-cell-based adaptive immune response on the other hand, in which they finally act as immunoeffector cells. Macrophages are also essential for tissue regeneration and remodeling. Cells of the monocyte–macrophage system showed impaired cytokine release [5,6], reduced oxidative burst [7,8], and cytoskeleton alteration [9] in microgravity, and massive changes in gene expression under altered gravity [10].

The underlying causes how gravity effects or regulates mammalian cells are still largely unknown. However, there are considerations of a direct mechanical effect on cell shape and geometry [11] that is transmitted from the cell membrane through the cytoskeleton and into the entire cell, including the nucleus [12]. In our previous work, we had already identified many effects of gravity changes in different cell types of the human immune system and detected effects in signal transduction cascades, cell cycle control proteins, regulation of adhesion molecules, and oxidative-burst response [13]. However, evidence for a conclusive cause of the regulation of cellular processes by gravity was not found in these earlier studies.

Then, we performed comparative studies of complete transcriptome analyses after different periods of altered gravity on parabolic flights and ballistic suborbital research rocket missions. Here, we found rapid and pronounced transcriptome changes in human Jurkat T cells after as little as 20s of altered gravity (microgravity as well as hypergravity), which was followed by a rapid and nearly complete adaptation response after 5 min [14,15]. In parallel, we identified extremely stable genes that were consistently expressed at all times under all gravity conditions [16,17]. Recently, we demonstrated in human Jurkat T cells that genes are differentially regulated in cluster structures (“gravity-responsive chromosomal regions”, GRCRs) under altered gravity. Combining this with high-throughput chromatin conformational analysis (Hi-C), we identified a highly significant association of GRCRs with structural 3D chromatin changes that colocalize mainly on the small chromosomes (chr16-chr22) [18].

However, our studies to date have not yet been able to show whether, in addition to the chromatin structure-specific transcript changes found, there are also changes in RNA polymerase II DNA binding or epigenetic changes. Additionally, so far, we have studied only T cell lines but not primary cells. We therefore chose the model system of isolated primary human macrophages not only because of its immunological relevance, but also to investigate whether basic structural transcriptional and chromatin-modifying effects are also detectable in other cell types.

All histones can be post-translationally modified, especially at histone tails, directly regulating chromatin structure and recruiting non-histone proteins to chromatin. High levels of H3K4me3 are found at the promoter sequences of active genes [19]. Trimethylation of lysine 4 on histone H3 (H3K4me3) is a nearly universal chromatin modification at the transcription start site of active genes in eukaryotes and reflects the extent of transcription. This association is thought to be both a cause and a consequence of transcription, indicating a close interaction between transcription and H3K4me3 [20,21]. Therefore, whole-genome examination of structural DNA-H3K4me3 binding may provide direct evidence of gravity changes on DNA-interacting chromatin architecture and epigenetic global changes in chromatin.

Based on these findings, we could postulate a general model of cellular gravisensing through changes in nuclear 3D structure [18]. Furthermore, recent transcriptomics results showed a nonrandom distribution of direction of differential gene expression over the chromosomes, which is aligned with such mechanism [22].

Whole-genome epigenetic detection of DNA-protein binding at different loci is possible using chromatin immunoprecipitation (ChIP) techniques. Since its invention in 2007, the ChIP-seq technique has rapidly become the leading technology for analyzing DNA-binding proteins and their binding sites [23]. In contrast to its widespread application, the formaldehyde-induced DNA–protein crosslinking still entails methodological issues [24]. For example, kinetics, interactions, and dependencies of protein conformations and the functionality of the detecting antibodies are poorly understood [25]. Additionally, highly dynamic protein–DNA interactions are likely to be undetectable by ChIP [26] and detection of interactions is highly dependent on the cellular environment [27], which may lead to “carryovers”, i.e., detection of events that occur after the onset of fixation. Of course, constraints and limitations are inherent to any method. In the case of ChIP, we accounted for these possible carryover effects in the experiment design and in the conclusions drawn from the data.

The experimental environment of a parabolic flight also imposes operational and thus methodological–experimental constraints. Essentially, this is the physically defined maximum time of the microgravity phase due to the parabolic maneuver performed in the Earth’s atmosphere. Therefore, also here it had to be judged whether the experiments should be better performed in “simulated microgravity” under laboratory conditions or under “real microgravity” under flight conditions. While operational constraints in real microgravity flight samples limits the level of maximum standardization, more and significant methodological limitations exist for ground-based simulations [28]: Previous studies of ground-based 0 g simulations using the random-positioning machine (RPM) showed significant shear forces [29,30], which can reach up to 100 mPa [31]. Additionally, the hypothesis that zero gravity corresponds a vector sum zero of acceleration force, may be true for a point particle, but not for an extended system [28]. Indeed, in our own previous experiments with human Jurkat T cells, less than 1% of the transcriptome changes in flight-induced microgravity could be reproduced by clinorotation [14]. For this reason, the existing ground-based simulations did not appear as a methodologically acceptable experimental basis for the systematic study of human cells in altered gravity.

In order to study an in vivo near system of immunologically relevant human cells under physically real microgravity, we performed parabolic flight experiments with primary human macrophages. The experiments were standardized to the highest possible degree, as far as the experimental limitations of the flight experiment and the ChIP-Seq method allowed.

## 2. Results

**Figure 1:**
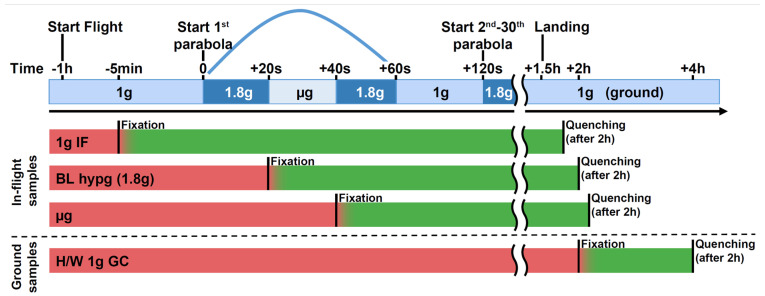
Experimental fixation scheme showing the different sample groups and their distinct fixation and quenching times. Sample groups 1g IF (in-flight), BL hypg (baseline hypergravity), and μg (microgravity) were flown onboard the parabolic flight aircraft. The 1g IF group was fixed 5 min prior to the first parabola by addition of formaldehyde, BL hypg was fixed at the end of the first hypergravity phase and μg at the end of the microgravity phase. The latter group therefore has been exposed to 1g in-flight, hypergravity and consecutively microgravity prior to fixation. To stop the chemical crosslinking, Glycine/PBS was added as quencher after 2 h of fixation, directly after landing and deboarding. The 1g GC (ground control) reference was not included in the flight and was fixed shortly after landing and quenched after two hours. Red bars indicate active biological samples, green samples that undergo crosslinking. A potential carryover (“smearing”) is indicated by a red-green color gradient. This could occur due to slow crosslinking kinetics, where effects happening after the beginning of fixation are still recovered if cellular dynamics are not stopped by formaldehyde immediately.

The aim of this study was to assess the binding patterns of the regulatory histone H3K4me3 and of RNA polymerase II in human immune cells during short phases of microgravity to test if epigenetic reactions towards hyper-/microgravity already appear after a few seconds of stimulation. Therefore, primary human cells have been exposed to 20 s of hypergravity (BL hypg) and consecutive 20 s of microgravity (μg) during the 28th DLR parabolic flight campaign (PFC), and fixed at different points in time to yield different gravity condition groups (Figure 1). Samples consisted of primary human macrophages isolated from donor blood from six donors to provide an in vivo near system of immunologically relevant human cells.

To analyze the binding behavior of RNA Polymerase II and histone H3K4me3, areas in the genome were identified that were enriched for reads after performing ChIP sequencing and therefore representing areas of protein–DNA interaction. These ChIP-seq peaks have been called out of raw RNA fragment reads, consensus peak sets have been defined, and consecutively, ChIP peaks with significant change in binding strength between different conditions of gravity have been identified and classified.

**Figure 2:**
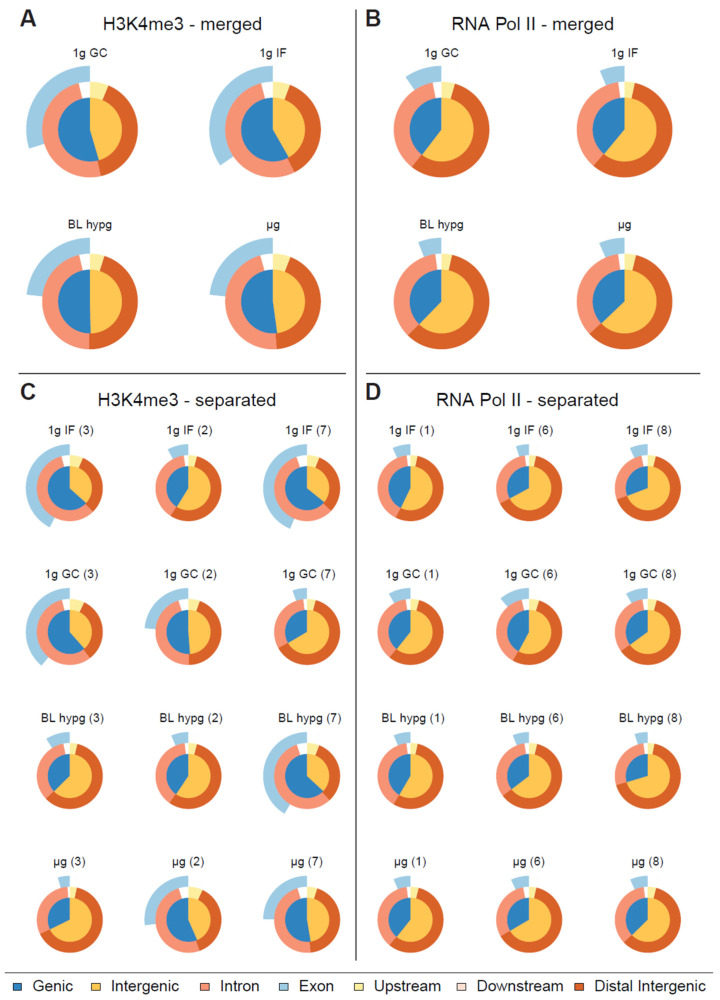
Classification of peaks called by MOSAiCS displayed as Venn pie diagram for different gravity conditions. The inner circle represents the fraction of genic and intergenic sequences, the outer circle rings represent the fractions of intronic/exonic sequences for genic sequences and for peaks upstream/downstream and distant from transcription start sites. (**A**,**B**) Called peaks for H3K4me3/RNA Pol II, replicates merged by gravity condition. No clear pattern emerges in terms of peak distribution along different gravity conditions for RNA Pol II, H3K4me3 might have a subtle reaction towards altered gravity by decreasing the ratio of genic peaks over intergenic peaks. (**C**,**D**) Classification of called peaks for H3K4me3/RNA Pol II, each sample displayed separately. Blood donor number is indicated in brackets.

To analyze the protein–DNA binding behavior under different gravity conditions, fixed, immunoprecipitated and processed samples (three per gravity condition per protein of interest) have been sequenced. From raw ChIP-seq reads (filtered for ENCODE blacklist regions) (Figure S1), binding peaks have been identified with the help of MOSAiCS peak caller (Figure S2), an R-based peak caller that natively supports sharp and broad peaks, which has been chosen to account for the property of RNA Pol II to display peaks in a broad range of sizes. For better comparability to H3K4me3 which is considered to form narrow peaks, RNA Pol II was initially also analyzed in sharp peak mode. For each gravity condition results of three individual blood donors were merged and a first comparison was performed on aggregated data, where identified peaks were categorized depending on their distance in relation to the closest genes as genic or intergenic, and exonic, intronic, upstream, downstream, or distal (Figure 2A,B). However, the differences between the four investigated gravity conditions were not significant in this analysis mode (Figure 2A,B). We therefore analyzed the samples individually and compared the results of the different blood donors within the same gravity condition (Figure 2C,D). For H3K4me3, pronounced inter individual differences could be identified (Figure 2C) These donor-specific effects still occurred despite cells having been cultivated under reproducible standard conditions for 10 days before the start of the experiment. This clearly visualized the large and persistent biological variability within primary, donor-derived immune cells, a challenge that will be faced during larger space missions. In contrast, the RNA Pol II samples did not show such blood donor dependent effects (Figure 2D).

**Figure 3:**
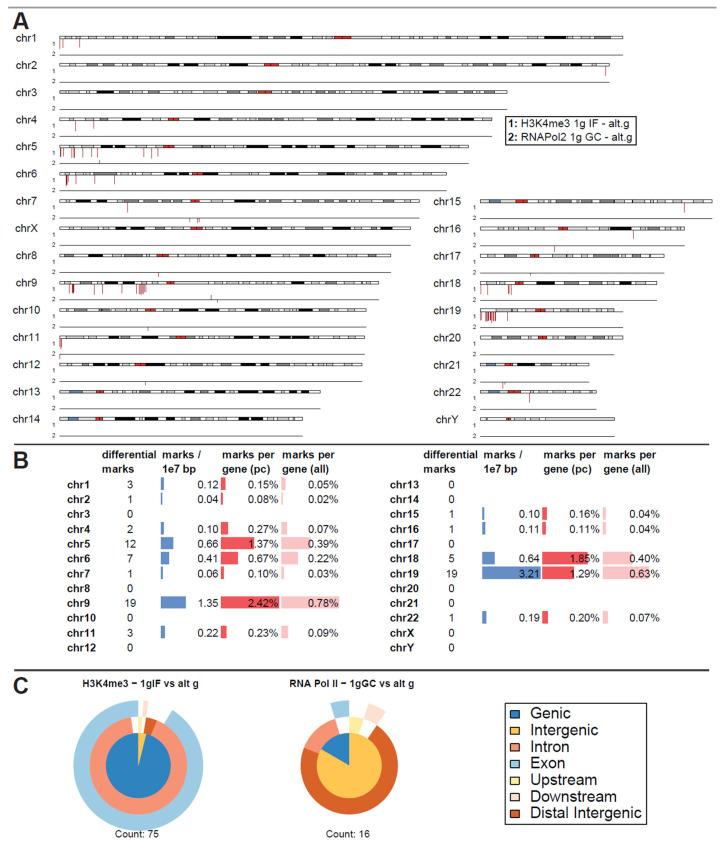
Differential peak binding between regular gravity (1g) and a merged altered gravity condition (alt.g, merged between BL hypg and μg) to account for phase overlap of the two altered gravity conditions due to potentially slow crosslinking. (**A**) Significant differential binding events (FDR < 0.1) were displayed along the human chromosome set. The first line indicates H3K4me3 1g IF vs. alt.g and the second line RNA Pol II 1g GC vs. alt.g. A red dash indicates weaker binding under altered gravity, a blue dash stronger binding. Lengths indicate relative magnitudes of changes in binding strengths. For other comparisons, no significantly differential sites could be identified. H3K4me3 showed only binding sites with lowered binding affinity that were mostly accumulated at distinct locations on only a few chromosomes (chr5, chr6, chr9, chr18, chr19). No clear pattern could be identified for RNA Pol II. (**B**) Quantification of the significantly differential marks for H3K4me3 from (**A**). Per chromosome, the number of marks, the density of marks per 10 million bp, the ratio of marks versus number of protein-coding (pc) genes, and versus number of all genes plus pseudogenes is given. (**C**) Classification of differential peaks by antibody for the comparisons. The total number of significant peaks is indicated below. In contrast to the distribution drawn from all called peaks that was mostly formed by intergenic peaks, the significantly different peaks for H3K4me3 only localized at intronic/exonic genic sequences. 00B3 0033 0037 ^7^.

The experimental flight hardware design did not allow an in-flight quenching procedure so that lower formaldehyde concentrations were required to avoid the risk of over-fixation. Considering potential fixation “smearing”, carryover of conditions that lie close to each other timewise, a first differential binding analysis was performed of normal gravity versus a joint altered gravity condition. Therefore, μg and BL hypg samples were merged, since BL hypg was fixed only 20 s before µg and therefore potentially “smeared” into this consecutive flight phase (Figure 1). A differential peak analysis was performed with the help of DiffBind, based on consensus peak sets (peaks present in at least three samples). If the number of reads of a certain peak statistically significantly differed from one condition to another, the peak was reported as significantly different, and a fold change could be calculated. Such significantly altered peaks could both be identified for H3K4me3 for the comparison of 1g in-flight versus altered g and for RNA Pol II for the comparison of 1g ground control versus altered g (Figure 3A), at a false discovery rate (FDR) threshold of <0.1 (corresponding to raw *p* values of 0.001 to 0.01 for H3K4me3 and 5 × 10^−6^ to 1.6 × 10^−3^ for RNA Pol II). For H3K4me3 1g ground control versus altered g and 1g in-flight versus altered g for RNA Pol II, no significantly altered peaks could be identified.

Remarkably, for H3K4me3, only peaks that showed weaker binding in altered gravity could be identified. Not a single peak with increased binding affinity was detectable (Figure 3A). Additionally, an unequal distribution was observable (Figure 3B): the four chromosomes chr5, chr6, chr9 and chr19 carried 57 of the 75 significantly differential peaks, all of them located on the p arm of the chromosomes. The density was exceptionally high for the relatively short chr19 with 3.21 differential peak marks per 10 million base pairs (bp), compared to an average of 0.24 over the entire genome. When comparing the number of marks to the chromosome’s gene density, chr9, chr19, chr5, partly again chr6, and additionally chr18 showed the highest ratios of significant peaks. The majority of these peaks have been classified as localized close to transcription start sites with most of them being close to both exonic and intronic sequences (Figure 3C). This pattern was clearly different from the overall distribution of H3K4me3 peaks from Figure 3B, even considering the large biological spread within the groups.

For RNA Pol II, only 16 seemingly uncorrelated peaks could be identified as differential, with no observable patterns in terms of increased / decreased binding (Figure 3A). Peak classification of these peaks was performed and yielded a pattern that was different from the one for raw peak calling. Since it only consisted of 16 peaks, a statistical interpretable distribution could not be assumed.

As a negative control, the contrast 1g GC versus 1g IF was assessed for significantly differential peaks for both antibodies, which should not contain any gravity-related peaks if fixation smearing would not affect the first parabola for the 1g IF condition. No significant peaks could be detected for H3K4me3 for a FDR of <0.1 (first peaks at FDR = 1.0) and only 2 significant peaks appeared for RNA Pol II for a FDR of <0.1 (8 peaks for a FDR < 0.5), one on chr2 and one on chr8. Therefore, the assay setup demonstrated stability against experimental noise induced by random biological effects and by the flight setup, which displayed a major source of technical effects for transcriptomics studies (Thiel, Hauschild et al., 2017).

Since crosslinking kinetics of formaldehyde are largely unknown and likely differ for every protein–DNA interaction, a split analysis was performed, where the two gravity conditions BL hypg and μg were analyzed separately, even though they had a temporal distance of only 20 s. Depending on the crosslinking behavior, this could improve the results if the hypergravity and the microgravity samples were so different, that an averaging, as performed above, would blur out singular effects. However, this could also compromise results if the samples from the two phases would be very similar, because then statistical power would be reduced by moving from six samples to three for the altered gravity condition.

**Figure 4:**
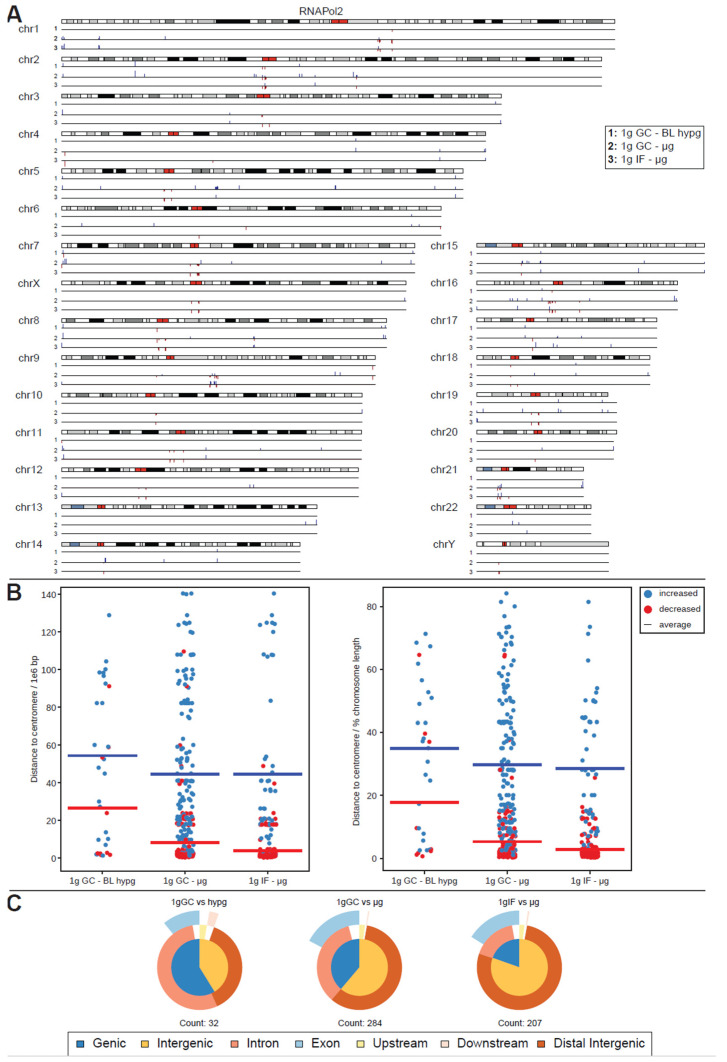
Differential peak binding between regular gravity (1g) and altered gravity conditions, split into BL hypg and μg to pinpoint effects observed for merged altered g, for RNA Pol II. (**A**) Significant differential binding events (FDR < 0.1) are displayed along the human chromosome set. Grey/black regions indicate dark cytobands, red regions indicate the centromeres. Line numbers indicate different comparisons of gravitational conditions. A red dash indicates weaker binding under altered gravity, a blue dash stronger binding. Lengths indicate relative magnitudes of changes in binding strengths. For other comparisons, no significantly differential sites could be identified. (**B**) Distribution analysis of differential peaks per comparison. The distance of a peak to the centromere of the corresponding chromosome is given, in absolute distance (left, 1e6 bp) and in relative units (right, percent of chromosome length). The averages per comparison is indicated by a line, stratified by direction of difference. (**C**) Classification of differential peaks by antibody for the comparisons. The total number of significant peaks is indicated below.

For RNA Pol II, this analysis yielded a surprise (Figure 4A). The number of significantly different peaks at FDR < 0.1 (corresponding to raw *p* values of 10^−28^ to 0.01) increased by an order of magnitude for comparisons to 1g ground control (284 peaks for 1g GC vs. μg and 32 peaks for 1g GC vs. BL-hypg), additionally the comparison of 1g in-flight to μg resulted in 207 significantly different peaks, where no differential binding sites could be identified for the merged comparison. A large fraction of peaks for 1g IF vs. μg was also present in 1g GC vs. μg with 1g GC vs. μg bearing additional peaks that were unique to this comparison. This behavior was expected, since under ideal conditions 1g IF vs. μg should contain only peaks that are specific to altered gravity, 1g GC vs. μg should contain the same peaks but bear additional ones associated with effects from the overall flight (takeoff, flight vibrations, etc.) that should be cancelled out in the 1g IF vs. μg comparison. Since the crosslinking kinetics might cover slowly crosslinking loci that are related to altered gravity in the 1g IF vs. μg comparison due to crosslinking carryover, it was still worthwhile to analyze both the 1g IF and the 1g GC comparison; 1g GC vs. BL-hypg showed 32 differential peaks, most of them shared with 1g GC vs. μg. These could be hypergravity-specific or carryovers from the microgravity phase; 1g IF vs. BL-hypg did not show any differential peaks at all.

The peaks for RNA Pol II were non-randomly distributed: sites of decreased binding strength localized close to the centromeres for most chromosomes, e.g., very prominently on chr2 or chr7. Sites with increased binding were spread out more evenly with a certain accumulation at the flanking regions of chromosomes. This observed behavior was quantified for all three comparisons (Figure 4B). The distance of significant peaks where plotted, split by direction of change, for all three contrasts, in absolute base pair distance (left) and in relative units in percent of the length of the harboring chromosome of a peak; 1g GC vs. μg and 1g IF vs. μg both showed a strong tendency towards decreased binding close to the centromere (average distance 8.3 million bp resp; 5.3% for 1g GC vs. μg and 4.0 million bp resp.; 2.8% for 1g IF vs. μg). Additionally, increased binding was more spread out with an average distance 44.6 million resp.; 29.7% for 1g GC vs. μg and 44.5 million resp.; 28.6% for 1g IF vs. μg. For 1g GC vs. BL hypg, decreased binding peaks were also more located close to the centromere in contrast to increased binding, albeit much less pronounced and with much less peaks in general.

RNA Pol II peak classifications (Figure 4C) did notably differ from their overall composition for 1g IF vs. μg, where the fraction of intergenic peaks was elevated, which was as accentuated for 1g GC vs. μg. This difference could either indicate that peaks that truly resulted from altered gravity mostly classify as intergenic (since 1g GC vs. μg also contained peaks that result from ground vs. airplane effects) or that loci with rapid crosslinking behavior were preferably present in intergenic regions and were therefore more robust vs. carryover.

**Figure 5:**
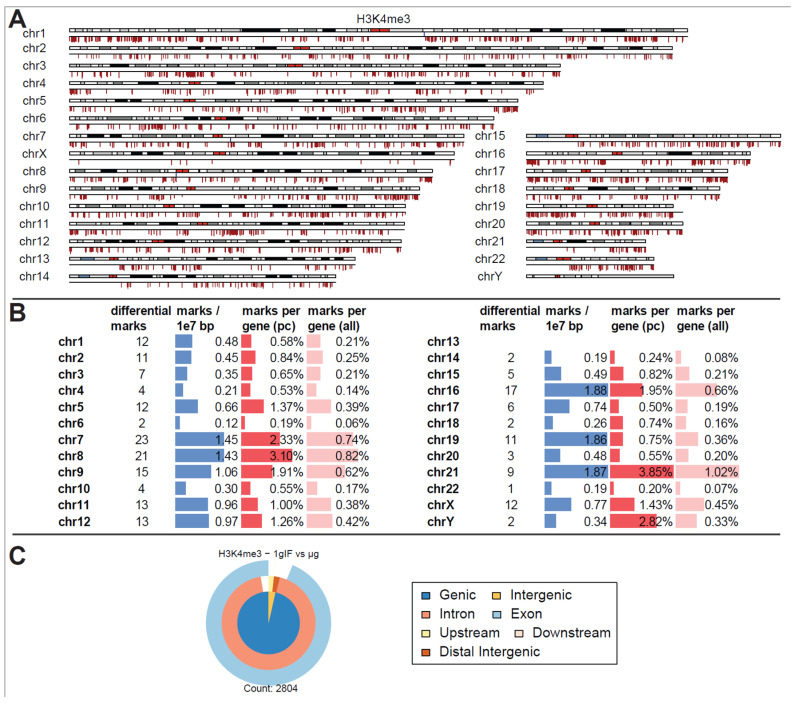
Differential peak binding between normal gravity (1g) and altered gravity conditions, split into BL hypg and µg to pinpoint effects observed for merged altered g, for H3K4me3. (**A**) Differential binding events (FDR < 0.31, lowest value present) for H3K4me3 1g IF vs. μg. Almost only peaks with decreased binding affinity in μg phase could be detected. Three sites of strong increase in binding could be found on chromosome 1, 7 and 8 close to the centromere. (**B**) Quantification of the significantly differential marks from (**A**). Per chromosome, the number of marks, the density of marks per 10 million bp, the ratio of marks vs. number of protein-coding (pc) genes, and vs. number of all genes plus pseudogenes are given. (**C**) Classification of differential peaks. The total number of significant peaks is indicated below.

In contrast to these findings, a separation into different altered gravity conditions led to a decrease in statistical power for H3K4me3 (Figure 5A). At a false discovery rate of <0.31 (corresponding to raw *p* values of 0.004 to 0.11 with a mean of 0.06), 2804 differential peaks emerged, among them only three peaks with increased binding strength could be identified (one on chr1 on the q arm, one on chr7 on the q arm, and one on chr8 on the p arm, all of them were located close to the centromere). Considering that each of the peaks taken on its own had a 30% chance of being a false positive this would qualify the separate results as a weak finding, but taken altogether, as for the aggregated analysis, an overwhelming fraction of sites showed decreased binding. Local clusters could again be identified but were more evenly distributed among all chromosomes (Figure 5B). Compared to the genome-wide average of 0.66 peaks per 10 million bp, chr16, chr19 and chr21 had an exceptionally high number of significant peaks of over 1.85. Compared to the chromosome’s gene density, chr21, and chr8 had the largest ratio of significant peaks. When classifying the peaks (Figure 5C), differential peaks for H3K4me3 mostly classified as genic in contrast to the overall composition of peaks for this antibody.

To test whether the results for RNA Pol II might be biased due to the application of MOSAiCS in sharp peak calling mode, the same analysis was performed for RNA Pol II with MOSAiCS in broad peak calling mode, using a Hidden Markov Model (HMM)-based calling algorithm. Since RNA Pol II peaks have a wide distribution in size, calling such peaks as sharp and broad is both legitimate. The previous results could be confirmed for peak type distribution (Figure S3), differential peak calling between conditions (with slightly elevated number of significant peaks) and geometrical distribution on the chromosomes (Figure S4) and the relatively small proportion of genic peaks for 1g IF vs. μg. Therefore, the previous results were stable under this peak calling model.

**Figure 6:**
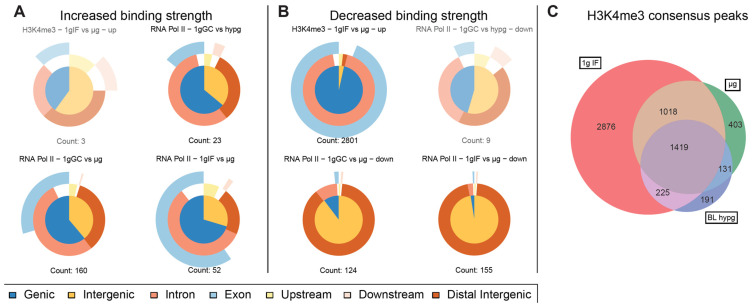
Characterization of significantly altered peaks from previous differential binding analysis. Separated by up- and downregulated peaks to visualize potential trends for the direction of regulation for the three comparisons for RNA Pol II and one comparison for H3K4me3 with significant differences. Vennpie diagrams that only include a number of peaks below ten are faded out since a robust analysis is not indicated if the number of peaks is too small. (**A**) Upregulated peaks only. Number of peaks indicated below. (**B**) Downregulated peaks only. Number of peaks indicated below. For RNA Pol II comparisons including μg, the downregulated peaks were almost exclusively characterized as distal intergenic peaks, upregulation was not as strongly skewed as downregulation but had a clear trend towards genic peaks. (**C**) Total number of peaks per condition of H3K4me3 that occurred in all three replicates of a condition and were therefore considered consensus peaks. Overlaps between conditions and unique peaks are indicated by the (non-) overlapping regions of the Venn diagram. Relative areas correspond to number of peaks.

Driven by the observation that peaks with significantly increased and decreased binding strength for RNA Pol II showed a fundamentally different distribution along chromosomes, separate analysis of such peaks was performed (Figure 6A,B). Such separation yielded robust results for RNA Pol II for both μg comparisons but not for the hypergravity comparison (and for H3K4me3) because the number of peaks with decreased (increased) binding fell into the single digit range which rendered comparisons inapplicable due to the risk of statistical shot noise. For the two microgravity comparisons, peaks with increased binding consisted of approximately 2/3 genic peaks (which classified mostly as intronic) and partly exonic for the ground control comparison (1g GC vs. μg), and mostly genic (intronic + exonic) for the flight comparison (1g IF vs. μg). The largest difference between both was the number of peaks showing increased binding which was more than 3× larger for the ground control comparison. This seems to explain why the genic fraction was larger for the ground control comparison in the overall analysis (Figure 4B). Peaks with decreased binding strength mostly consisted of intergenic sequences that were remote from any genes for these two comparisons (Figure 6B), which was in line with the observation that peaks with decreased binding were localized very close to or overlapping with the centromere.

Therefore, under microgravity, RNA Polymerase II showed increased binding to several loci that correspond to genes but decreased binding to central regions that do not correspond to genes.

For the histone, additionally to lowered binding strength, the overall number of consensus binding peaks within gravity conditions was investigated (Figure 6C). A Venn diagram was generated based on which peaks from consensus peak sets from the different gravity conditions did overlap. The number of peaks decreased from 1g in-flight to BL hypg and μg with a slight recovery from BL hypg to μg. Only a small number of peaks were unique to BL hypg and/or μg, most peaks of these two conditions were inherited from 1g IF. This result added additional evidence to the finding that H3K4me3 lost binding strength under altered gravity: not only the strength of the bindings but also the overall number of binding sites was decreased under microgravity compared to normal gravity.

**Figure 7:**
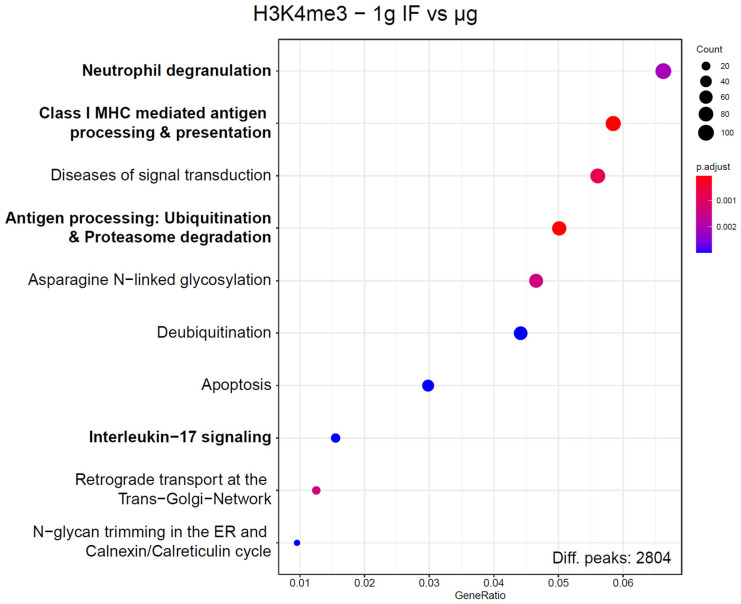
Enrichment analysis of genes that correspond to ChIP peaks with significantly altered binding strength for H3K4me3. Corresponding genes have been defined by having their transcription start site within 3000 bp of the ChIP peak maximum. Reactome pathways were screened for enrichment over the expected value based on a random distribution, 10 pathways with the highest gene ratio are displayed. Number of hit genes is indicated by circle sizes, fraction of hit genes compared to all genes in a set is given by the Y axis value. Adjusted *p* values are given by the color scheme. Immune-associated pathways are indicated in bold. These Reactome pathways share no to almost no genes with each other, with the exception of “Antigen processing”, which has a significant overlap with “Class I MHC mediated antigen processing & presentation”.

The large number of sites with differentially bound peaks for H3K4me3 under microgravity almost exclusively consisted of genic regions. To test if these hits were randomly distributed or followed a certain systematic, an enrichment analysis exploiting the Reactome database was performed (Figure 7). Since these enrichment analyses would always produce hits if only a large enough set of genes would be supplied, a strict corrected *p* value cutoff of <0.01 was applied. For this dataset, out of the 10 most enriched pathways, 4 immune-associated pathways have been identified. The pathway with the highest gene ratio was “Neutrophil degranulation” with 111 genes out of 2804 total peaks downregulated in the dataset, out of the 480 genes that are part of this pathway. These pathways have been assessed for overlaps, only “Class I MHC mediated antigen processing & presentation” and “Antigen processing: Ubiquitination & Proteasome degradation” had a significant overlap of included genes. These four pathways contained 220 unique hits from the sites of interest derived from the significantly different peaks, out of the 2804 total peaks. They contained 952 unique protein-coding genes. With a Fisher’s exact test *p* value of 4.7 × 10^−16^, this displayed a highly non-random distribution. Since multiple independent immune system pathways displayed the largest group of associated pathways of H3K4me3 binding changes under microgravity and represented almost 10% of all hits, a functional association with the immune system seems likely.

## 3. Discussion

Regarding the validity of the methods used, the experimental system was shown to be robust to random biological effects due to the flight experiment, which is typically a major source of technical confounding effects in transcriptomics studies [15].

Due to the operational and technical limitations of the flight experiment, quenching had to be performed after landing. Therefore, an experimental approach with a relatively low formaldehyde concentration had to be chosen. Since little data are known to date about the specific timing of the biochemical crosslinking reaction in different environments, it is possible that the crosslinking processes are not completely finished at the end of the respective flight phase. Such an effect could be present in the H3K4me3 detection, as no differences could be measured between the 1g vs. BL hypg groups. In contrast, when the BL hypg and μg groups, which were only 20 s apart, were merged in comparison to the 1g group, significant differences could be detected. In the case of RNA Pol II detection, the “carryover” effect seems to have less impact, as separation into a hypergravity and a microgravity phase increased statistical significance, and clear differences between all comparisons could be observed. The complex kinetics of formaldehyde cross-linking poses an inherent experimental challenge that must therefore be considered when interpreting the results.

In this study, primary human M1 macrophages from blood donors were used, which a priori suggests some biological variance. In fact, the sample differences in H3K4me3 detection were clearly due to the different donors and were still present when the cells had been cultured under reproducible standard conditions for 10 days before the start of the experiment. In contrast, the samples showed no such interindividual differences in RNA-Pol II detection, indicating clear epigenetic differences but fewer differences in RNA-Pol II-dependent transcription between individual donors.

Interestingly, only downregulation of DNA-H3K4me3 binding loci could be identified in microgravity, which were chromosomally and structurally unequally distributed: Three-quarters of all affected loci were located on the four chromosomes chr5, chr6, chr9 and chr19, and there on the p arm of the chromosome. Here, the density for the relatively short chr19 was exceptionally high, with 3.21 differential peak marks per 10 million base pairs (bp), compared to an average of 0.24 across the entire genome. Most of the affected loci were localized near transcription start sites, with most of them located near both exon and intron sequences (Figure 3C).

The near-universal chromatin modification tri-methylation of lysine 4 on histone H3 (H3K4me3) at the transcription start site of active genes in eukaryotes reflect the amount of transcription [19,20,32] and is assumed to have an instructive role in transcription, while at the same time it is considered as the result of transcription [20]. The genomic distribution of the downregulated DNA H3K4me3 loci corresponds to a substantial extent to immunoregulatory genes. Due to the functional complexity of the affected gene groups and their interaction in the overall immunological system, further interpretations beyond this phenomenon are not possible here. Interestingly, in the affected degranulation pathway, indirect evidence for functional effects nevertheless exists, such as reports of reduced monocyte degranulation monocytes in astronauts after returning from a 5–11 day space flight [33], as well as in human natural killer cells after 12 h of simulated microgravity [34]. Thus, functional effects of microgravity on degranulation mechanisms could have an epigenetic cause.

RNA polymerase II is the main complex of the transcription apparatus [35]. In microgravity, analysis of DNA-RNA polymerase II binding showed increased binding to multiple loci at coding sequences but decreased binding to central noncoding regions. Detection of altered DNA binding of RNA polymerase II provided direct evidence that gravity changes can lead to altered transcription. Gravity changes led to the detection of loci with both increased and decreased RNA polymerase II binding strength. Here, loci with decreased binding strength were found in centromere–proximal regions of noncoding regions of the genome, whereas loci with increased binding strength were found in coding regions distributed throughout the chromosome. These findings are in full agreement with the downregulation of the transcript pool after 20s microgravity found in both Jurkat T cells and myelomonocytic U937 cells [10,14,15].

The detected whole-genome effects of gravity-induced changes on DNA-H3K4me3 and DNA-RNA polymerase II binding loci are in agreement with the gravitational genomic model developed on Jurkat T cells [18], according to which ultrafast transduction [36] of mechanical forces across the cytoskeleton to the nucleus and chromatin [37,38] alters the spatial configuration of chromatin, thereby affecting gene ex pression [39,40]. This model is based on the assumption of a “mechano-genetic code” for which a wide range of experimental evidence exists [39,41,42,43].

We recently found in real-time experiments on a suborbital ballistic research rocket that the cytoskeleton of primary human M1 macrophages responded to gravity changes within seconds and re-adapted within minutes [44]. In addition to the cytoskeleton, other cellular structures such as the whole cell membrane, nuclear membrane, and actin within the nucleus are likely involved in gravity-triggered changes. Since small forces in the low piconewton range can still trigger mechanotransduction in the nucleus, it is possible that the altered gravitational force is sensed and transmitted through the cellular [45,46] and the mechanical architecture of the nucleus [47,48,49]. This mechanotransduction induced by altered gravity could then alter nuclear plasticity, chromatin organization and accessibility, and subsequently gene expression [50,51,52,53], explaining how gravitational force—acting non-specifically on the whole cell—can cause such highly specific responses at the level of gene expression. Macrophages—a tissue-resident and at the same time highly dynamic cell population—are affected not only by nonspecific gravity on the whole cell but also by specific cell–cell and cell–matrix interactions, which contribute to the spectrum of mechanical forces acting via specific surface molecular structures such as integrins, focal adhesions, and more. Since in the case of gravity perception the whole cell would act as a sensor, whereby in the case of cell–cell and cell–matrix interactions spatially well-defined surface structures are involved, a difference in biological signal processing could be assumed. In our study, however, we are at the very beginning, have first described a basic cellular response, with all inherent limitations, and are not able to contextualize the processes at the tissue level or even at the level of the organism. Furthermore, our study is not able to address the fundamental question about the biological response to different doses of reduced gravity but can only describe a biological principle under the experimental conditions of a parabolic flight. A related International Space Station (ISS) experiment to study whole-genome gene expression and epigenetic changes in primary human M1 macrophages under different gravity conditions completely failed in 2018 due to a hardware malfunction on orbit (STaARS BioScience-8-Gene Control Prime-EPICON experiment).

Based on this study, we hypothesize that the rapid transcriptional response to changing gravitational forces is specifically encoded in the epigenetic organization of chromatin. Whether and how this gravitational-force-responsive coding is to be understood in terms of a coordinated adaptive response to altered gravity must be revealed by further functional studies.

## 4. Materials and Methods

### 4.1. Preparation of Biological Samples

Peripheral blood monocytic cell (PBMCs) were isolated from buffy coats from six different donors, provided by the blood donation Zurich service (Swiss Red Cross, Zurich, Switzerland) via Ficoll density gradient centrifugation. M1-Macrophages were differentiated according to the standardized Promocell^®^ method C-28055, where cells were subjected to a cytokine-driven differentiation process that lasted 10 days and comprises regular medium changes of M1 Macrophage generation medium DXF. Cells of 112.5 × 10^6^ were seeded per bottom of a standard 75 cm^2^ cell culture flasks, which matches the flight hardware dimensions. For the parabolic flight culture flask bottoms with the attached× M1 macrophages were put into 200 mL Nutrimix-bags (2112145, Braun, Kronberg, Germany) with 70 mL of medium, and subsequently integrated into custom made hard plastic containers as secondary containment. Samples were cultured and prepared for the flight in the laboratory at University of Zurich and transported to Bordeaux providing constant incubation at 36.5 °C before each flight day.

### 4.2. Parabolic Flight Experiments

Experiments under real altered gravity were performed during the 28th DLR Parabolic Flight Campaign (PFC) onboard the Airbus A310 ZERO g (F-WNOV) starting from Bordeaux airport (BOD). Cell culture flask bottoms inside their respective containers were stored inside a 36.5 °C incubator installed inside the aircraft. Samples were flown on campaign flight day 1 (1 March 2016), flight day 2 (2 March 2016), and flight day 3 (3 March 2016).

The parabolic flight included 31 consecutive parabolas, each consisting of a steady 1g flight phase, a hypergravity pullup phase of ~20 s, a microgravity phase of ~20 s and a second hypergravity phase of ~20 s. The experimental setup consisted of four different conditions, each with 6 replicate samples, one per donor number. The 1g inflight samples were fixed five minutes before the first parabola by addition of formaldehyde. The baseline hypergravity (BL hypg) samples were fixed at the end of the first hypergravity phase of the first parabola, and the microgravity sample (μg) was fixed at the end of the microgravity phase of the first parabola. Ground control samples did not board the plane but were stored under the same conditions and fixed right after the landing of the aircraft. A detailed experimental fixation scheme can be found in Figure 1.

### 4.3. Sample Crosslinking and Processing

Formaldehyde fixation was performed by injecting 7 mL of 0.5% formaldehyde via a syringe and connecting tubes, to the cells cultivated in 70 mL cell culture medium. To avoid over-fixation, Glycine/PBS in molar excess was added as a quencher after 2 h. The concentration had to be chosen lower than typical ChIP crosslinking concentrations to avoid over-fixation since the addition of a quencher was not possible in-flight due to hardware/regulatory constraints. Feasibility of chosen concentrations were determined by ChIP pre-studies in cooperation with ActiveMotif. Due to the largely unknown crosslinking kinetics of formaldehyde, a carryover effect from the first seconds of the subsequent parabolic flight phases cannot be excluded (illustrated in Figure 1).

After the flights, fixed cells were scraped of the culture flasks bottoms. Following, cells were washed once with PBS/Igepal and once with PBS/Igepal/PMSF and finally cells were pelleted and stored at −80 °C until further analysis.

### 4.4. Chromatin Immunoprecipitation and Sequencing

Samples were subjected to chromatin immunoprecipitation at the Core Facility Genomik, Medical Faculty of Muenster, Germany. The analysis was performed using ChipIT Express kit (ActiveMotif) according to manufacturer’s protocol using 100 μL chromatin and 3 μg antibody. H3K4me3 (39159, Active Motif, Carlsbad, CA, USA) and RNA Pol II (Cat No: ab5095, Abcam, Cambridge, UK) were selected as targets. Sequencing of DNA fragments was performed following a standard Illumina sequencer protocol. All 24 samples where sequenced on the same flow cell with a read depth of 25 million reads and 75 cycles.

### 4.5. Data Preprocessing

Sequence reads were demultiplexed by the proprietary Illumina bcl2fastq software. Raw FASTQ files where adapter-trimmed and quality-trimmed by TrimGalore [54] utilizing Cutadadapt and FastQC with standard settings but with a stringency setting of 3. The pipeline truncated low quality reads with a Phred score under 20 from the 3′ end, truncated the 13 base pairs Illumina standard adapters, and removed reads that became shorter than 20 bp. Alignment against the reference genome hg19 was performed using the STAR aligner.

### 4.6. Peak Calling

To account for the property of RNA Pol II to display both broad and sharp peak behavior, MOSAiCS peak caller was utilized [55] Mappability score, GC base content, and sequence ambiguity score from ENCODE were used for local peak thresholding. Sharp peak calling for RNA Pol II and H3K4me3 was performed using a background estimated method of moment model fitting with a fragment size of 200 (that has been cross-validated by signal convolution analysis) and a two-signal component model for peak determination at a false discovery rate (FDR) of 0.05. For RNA Pol II, broad peak calling was performed utilizing a hidden Markov Model (HMM) as model fitting and peak determination approach.

### 4.7. Peak Annotation and Classification

Peaks were associated with specific genes if the peak maximum lied within 3000 bp upstream or downstream of a transcription start site (TSS). Since RNA Pol II does not behave like a TSS-acting transcription factor, a peak was also associated with a gene if its maximum lied within any exon/intron of such gene even if this was more than 3000 bp downstream of the TSS. Based on these settings, annotation was performed using the ChIPSeeker function annotatePeak [56].

### 4.8. Identification of Differentially Binding Peaks

Significantly bound differential peaks were identified using the DiffBind package [57]. Raw peaks were filtered with a quality score cutoff of 20, assessed for shared peaks between samples and transformed into a consensus peak set that only contains peaks that were called in at least three samples. Consecutively, quantification and differential binding estimation of consensus peaks was performed by read counting using summarizeOverlaps, ignoring duplicate reads, and differential analysis with DESeq2, all based on aligned reads. Peaks with differential binding were reported that were below a defined FDR of 0.1 if not stated differently. Standard gene set enrichment analysis was performed against the Reactome database, using genes associated with significant differential ChIP peaks, as indicated above.

## Data Availability

The datasets generated during and/or analyzed during the current study are available in the GEO (Gene Expression Omnibus) repository (www.ncbi.nlm.nih.gov/projects/geo; accessed on 26 December 2022), accession no. GSE221397.

## Supplementary Materials

The following supporting information can be downloaded at: https://www.mdpi.com/article/10.3390/ijms24010603/s1.

## Abbreviations

BL	baseline
ChIP	chromatin immunoprecipitation
DLR	German Aerospace Center
FDR	false discovery rate
GC	ground control
GEO	Gene Expression Omnibus
HMM	hidden Markov model
IF	in-flight
MHC	major histocompatibility complex
PBMC	peripheral blood mononuclear cell
PFC	parabolic flight campaign
TSS	transcription start site
