# Supplementary material for: Rapid Downregulation of H3K4me3 Binding to Immunoregulatory Genes in Altered Gravity in Primary Human M1 Macrophages
Source: Int J Mol Sci. 2022 Dec 29;24(1):603. doi: 10.3390/ijms24010603 (PMC9820304; doi:10.3390/ijms24010603)
Supplement: Supplementary file 1 [file ijms-24-00603-s001.zip › Figure S2 - Called Peaks.pdf]

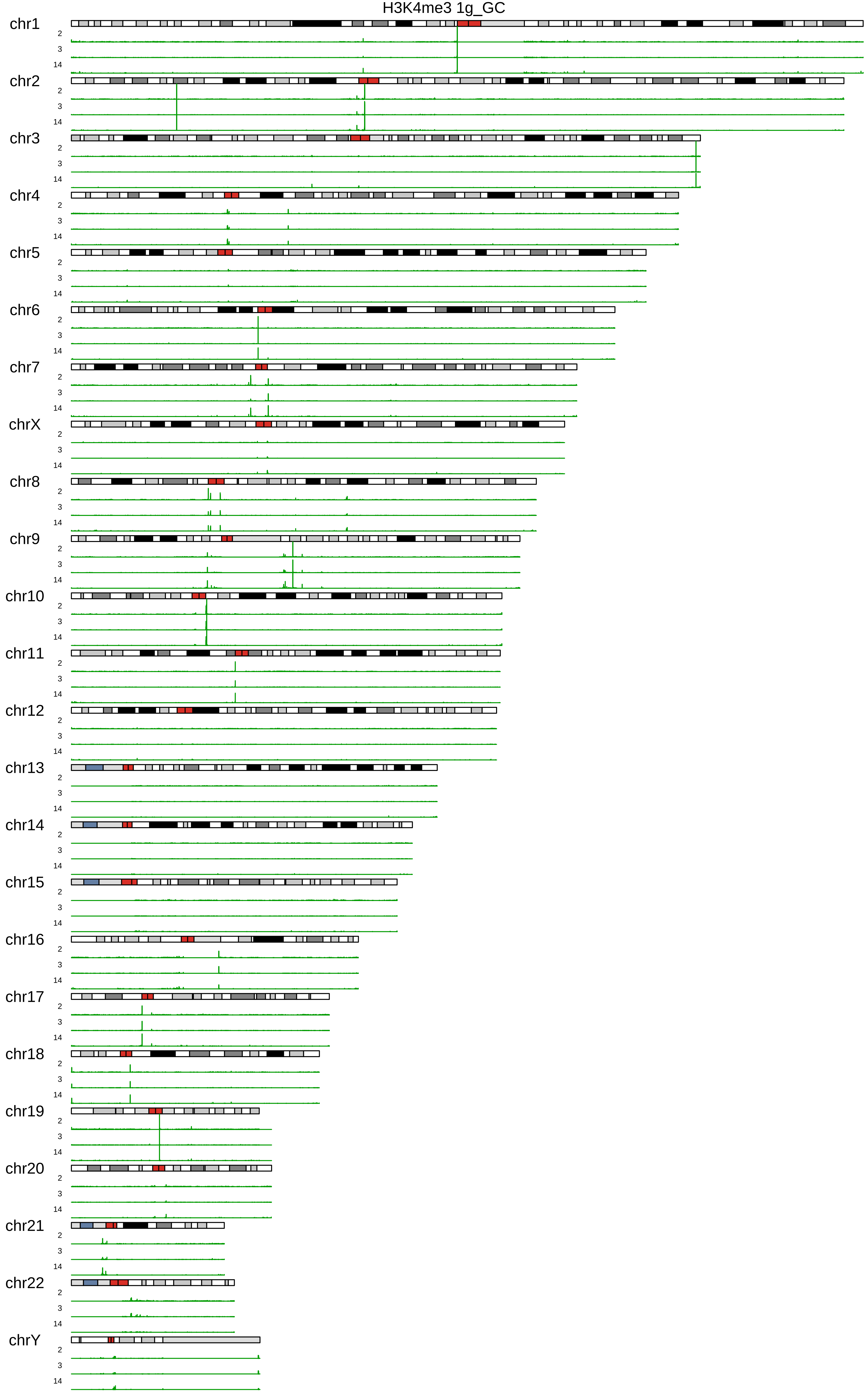

H3K4me3 1g_GC
chr1
chr2
chr3
chr4
chr5
chr6
chr7
chrX
chr8
chr9
chr10
chr11
chr12
chr13
chr14
chr15
chr16
chr17
chr18
chr19
chr20
chr21
chr22
chrY
2
3
14

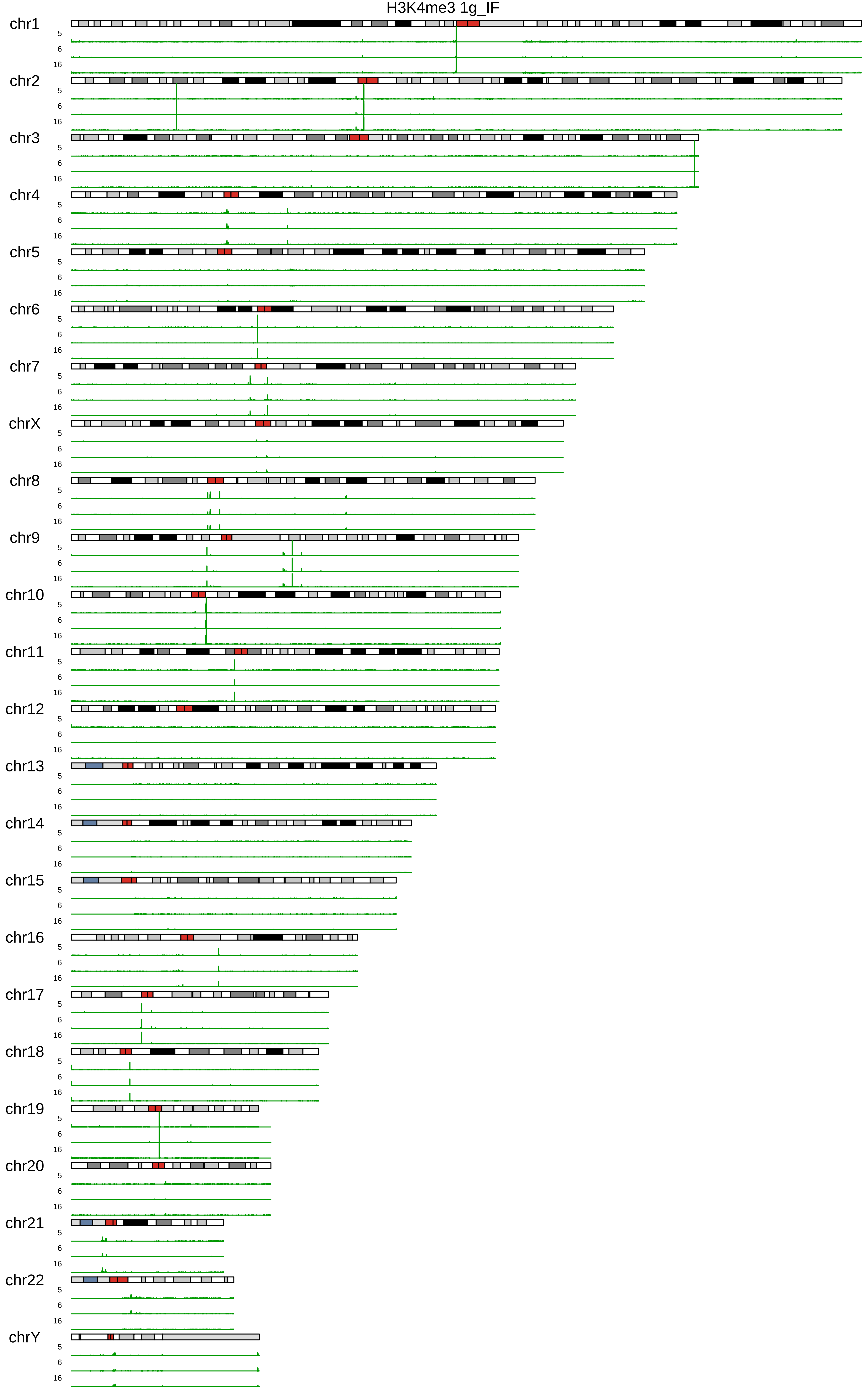

H3K4me3 1g_IF
chr1
chr2
chr3
chr4
chr5
chr6
chr7
chrX
chr8
chr9
chr10
chr11
chr12
chr13
chr14
chr15
chr16
chr17
chr18
chr19
chr20
chr21
chr22
chrY
5
6
16

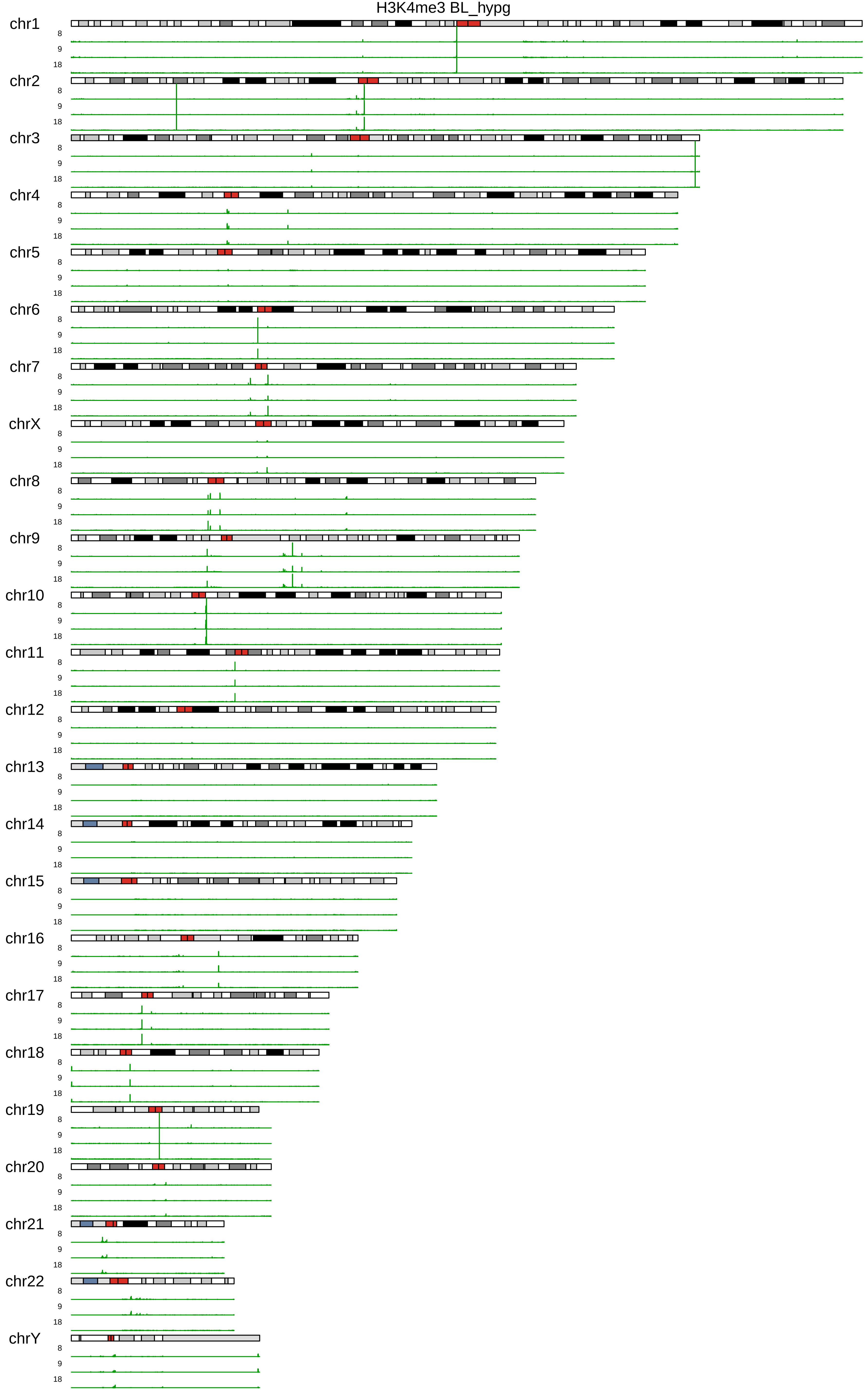
H3K4me3 BL_hypg
chr1
chr2
chr3
chr4
chr5
chr6
chr7
chrX
chr8
chr9
chr10
chr11
chr12
chr13
chr14
chr15
chr16
chr17
chr18
chr19
chr20
chr21
chr22
chrY
8
9
18

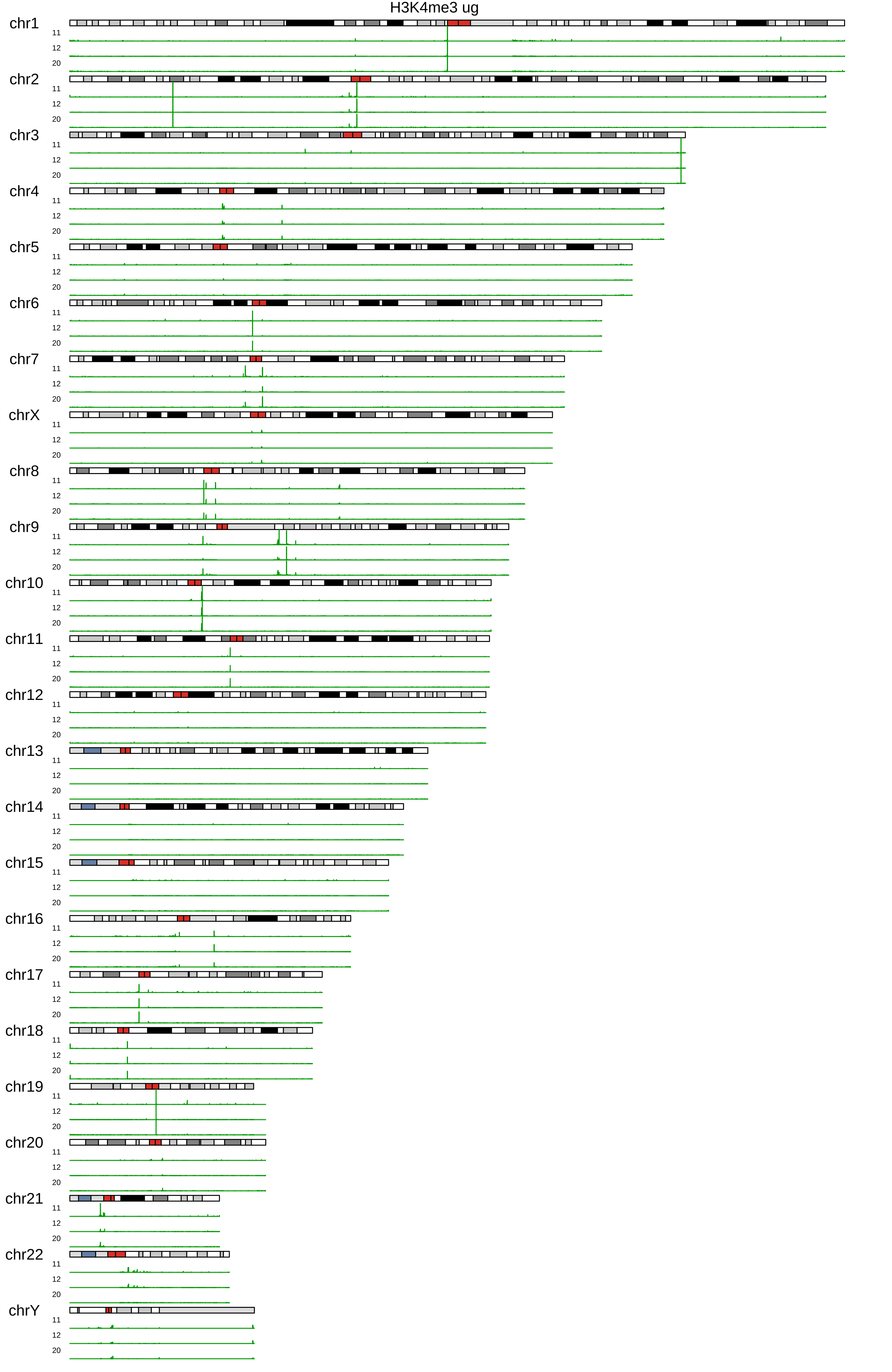
H3K4me3 ug
chr1
chr2
chr3
chr4
chr5
chr6
chr7
chrX
chr8
chr9
chr10
chr11
chr12
chr13
chr14
chr15
chr16
chr17
chr18
chr19
chr20
chr21
chr22
chrY
11
12
20

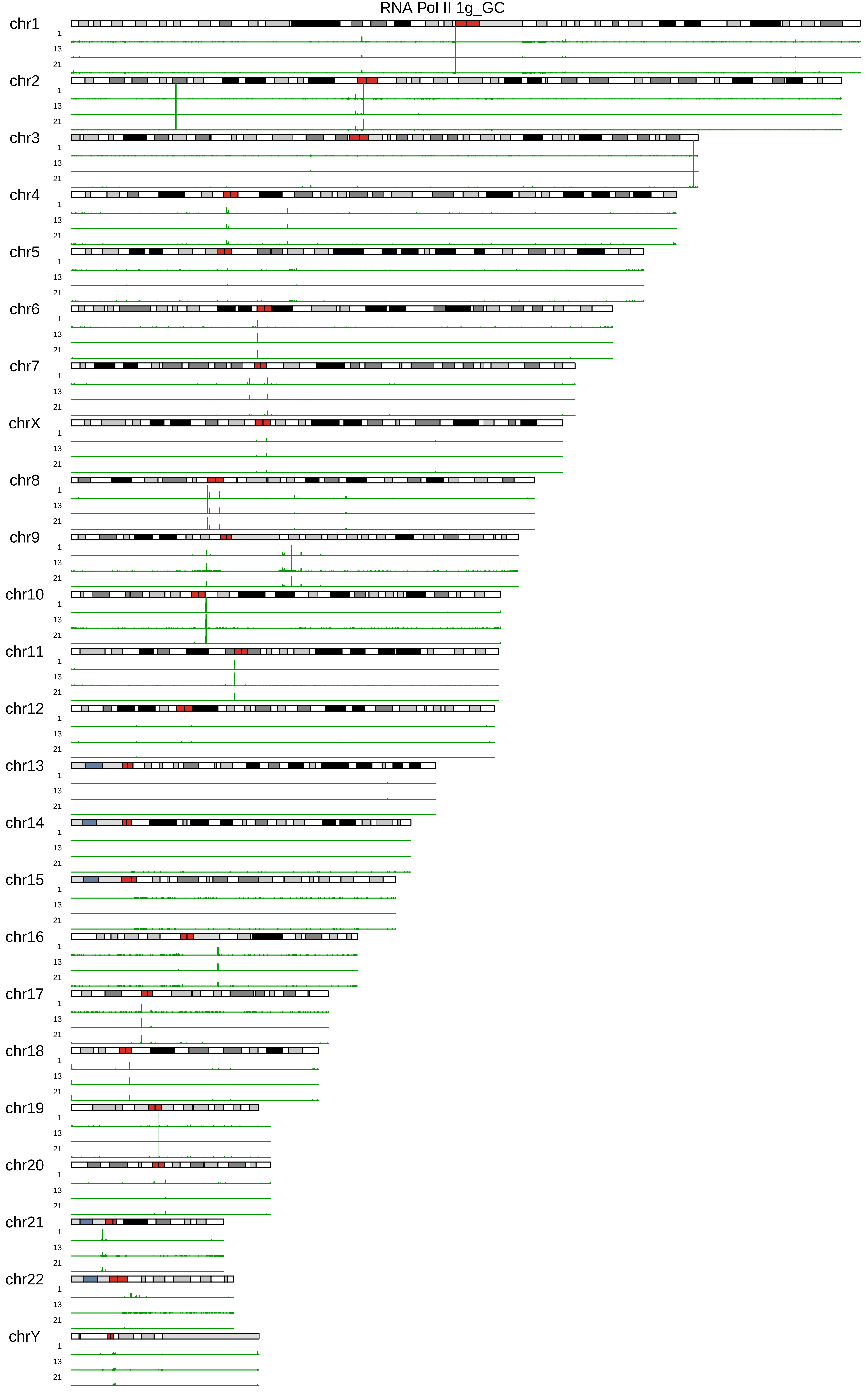
RNA Pol II 1g_GC
chr1
chr2
chr3
chr4
chr5
chr6
chr7
chrX
chr8
chr9
chr10
chr11
chr12
chr13
chr14
chr15
chr16
chr17
chr18
chr19
chr20
chr21
chr22
chrY
1
13
21

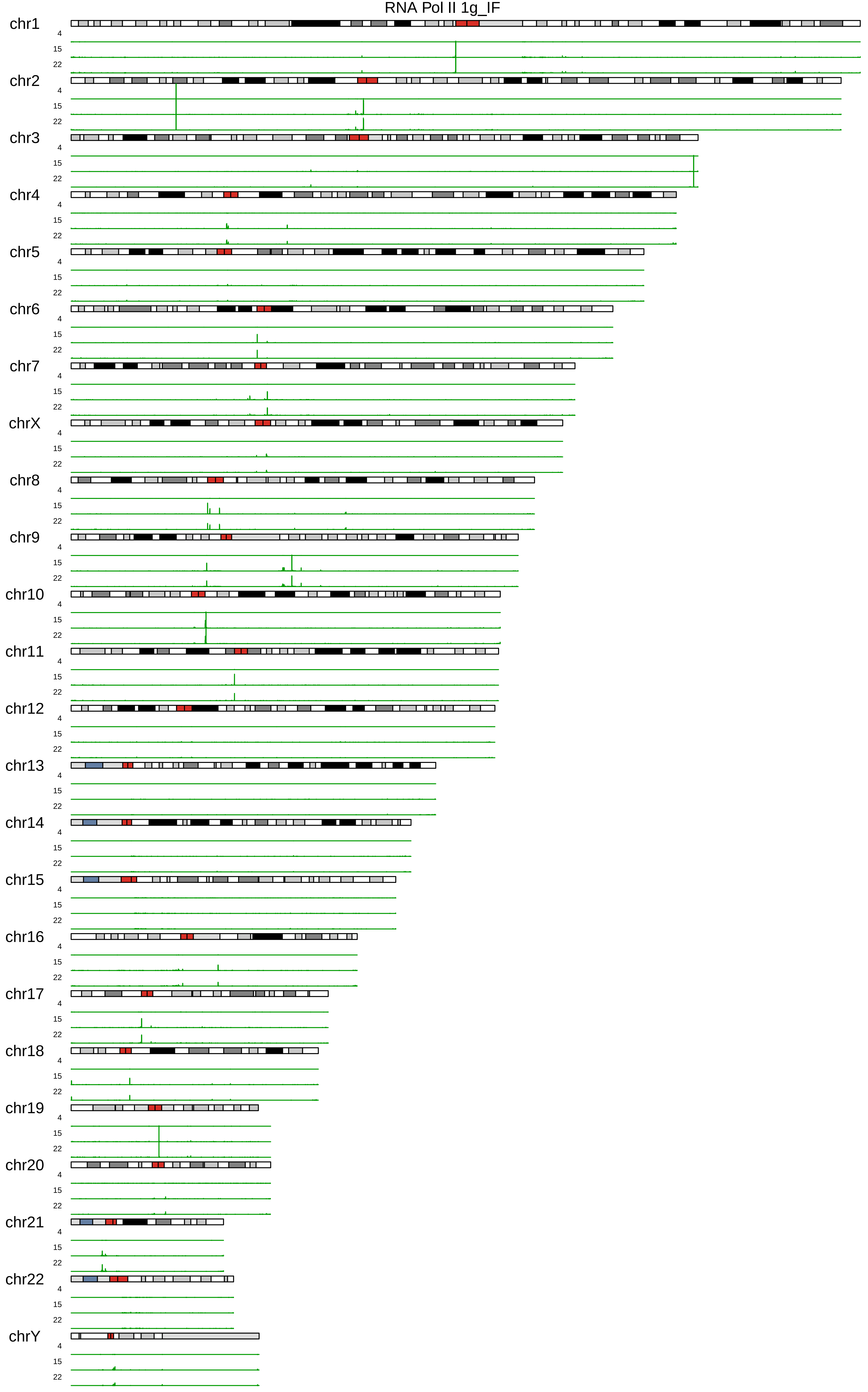
RNA Pol II 1g_IF
chr1
chr2
chr3
chr4
chr5
chr6
chr7
chrX
chr8
chr9
chr10
chr11
chr12
chr13
chr14
chr15
chr16
chr17
chr18
chr19
chr20
chr21
chr22
chrY
4
15
22

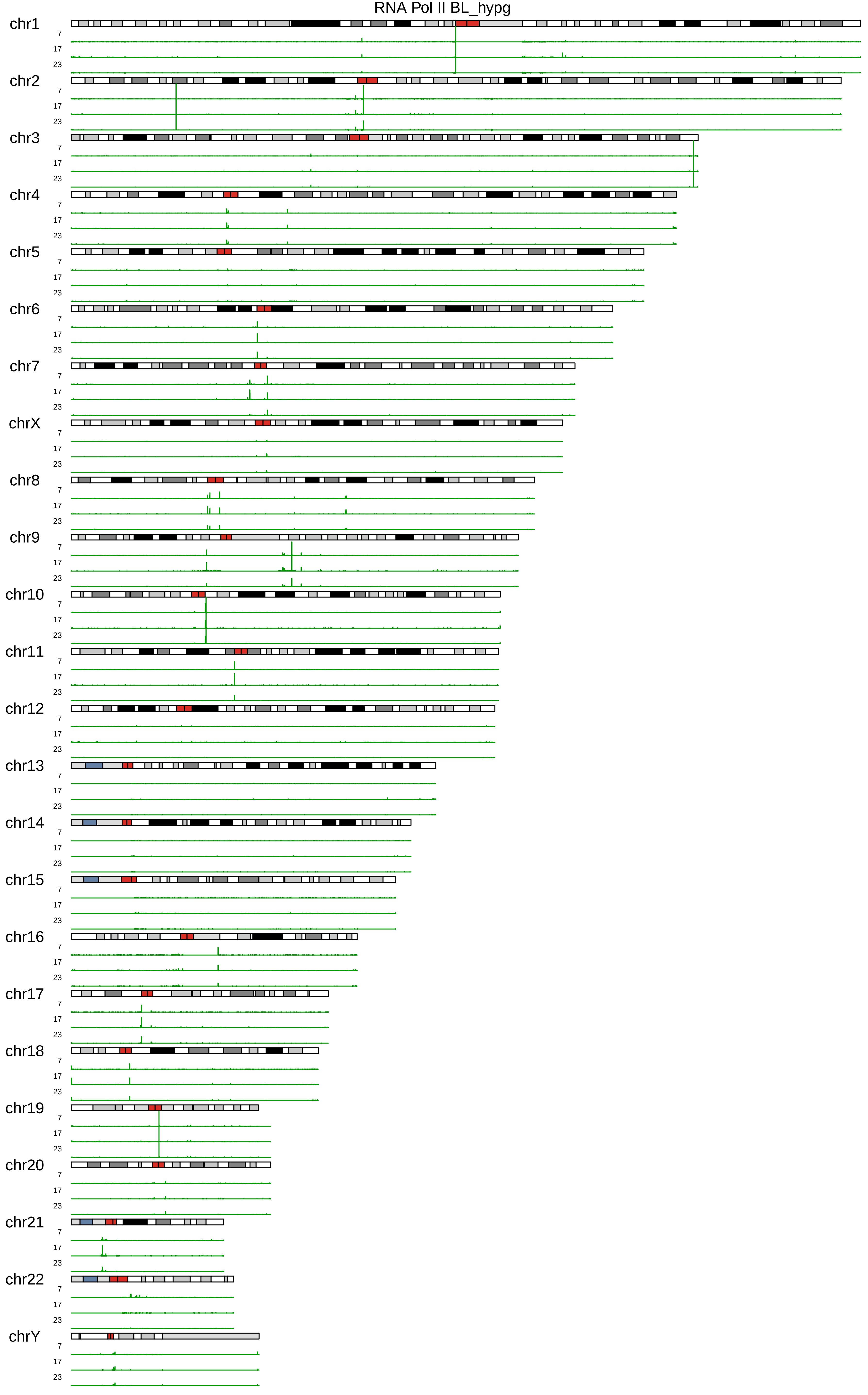
RNA Pol II BL_hypg
chr1
chr2
chr3
chr4
chr5
chr6
chr7
chrX
chr8
chr9
chr10
chr11
chr12
chr13
chr14
chr15
chr16
chr17
chr18
chr19
chr20
chr21
chr22
chrY
7
17
23

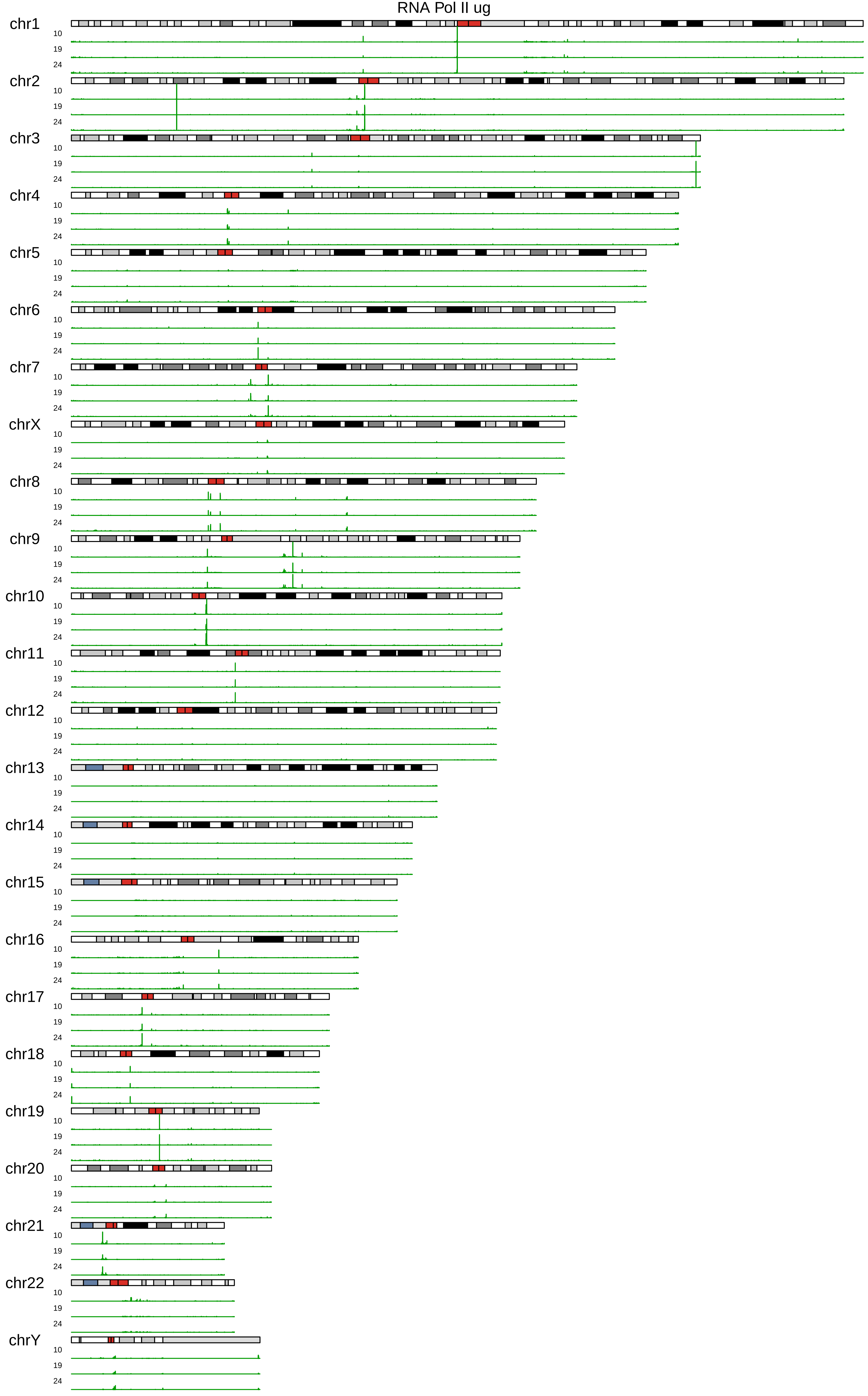

RNA Pol II ug
chr1
chr2
chr3
chr4
chr5
chr6
chr7
chrX
chr8
chr9
chr10
chr11
chr12
chr13
chr14
chr15
chr16
chr17
chr18
chr19
chr20
chr21
chr22
chrY
10
19
24

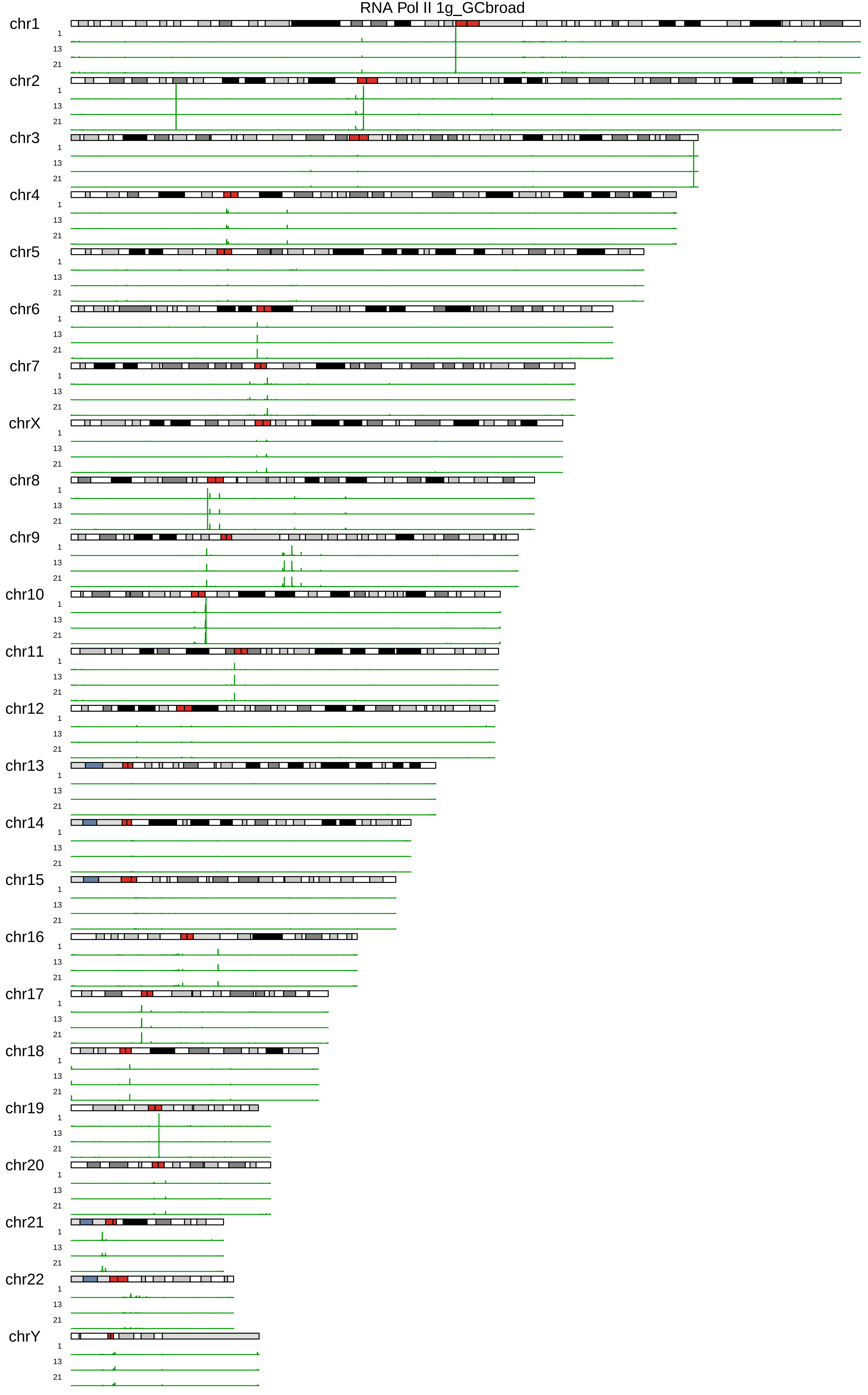
RNA Pol II 1g_GCbroad
chr1
chr2
chr3
chr4
chr5
chr6
chr7
chrX
chr8
chr9
chr10
chr11
chr12
chr13
chr14
chr15
chr16
chr17
chr18
chr19
chr20
chr21
chr22
chrY
1
13
21

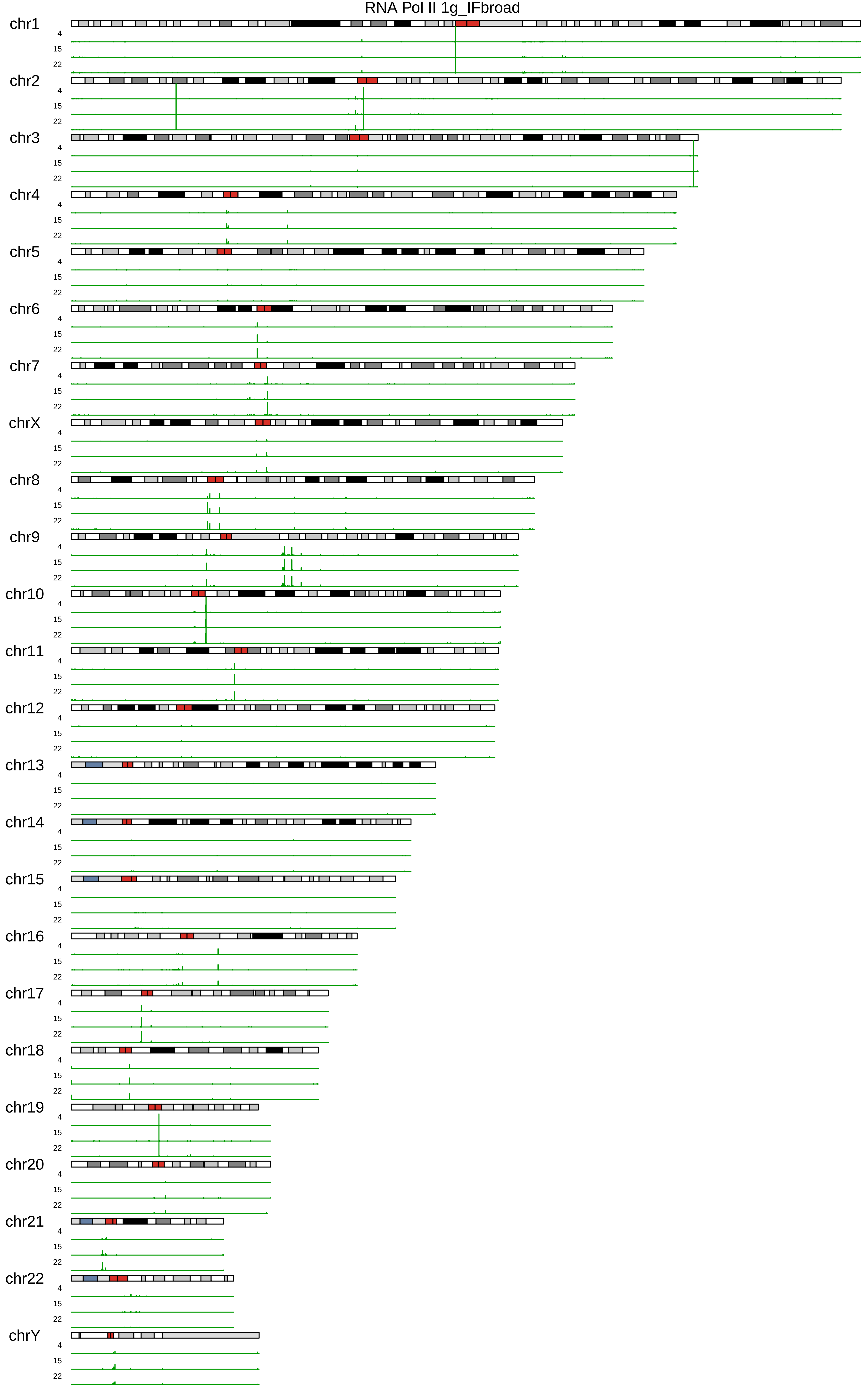
RNA Pol II 1g_IFbroad
chr1
chr2
chr3
chr4
chr5
chr6
chr7
chrX
chr8
chr9
chr10
chr11
chr12
chr13
chr14
chr15
chr16
chr17
chr18
chr19
chr20
chr21
chr22
chrY
4
15
22

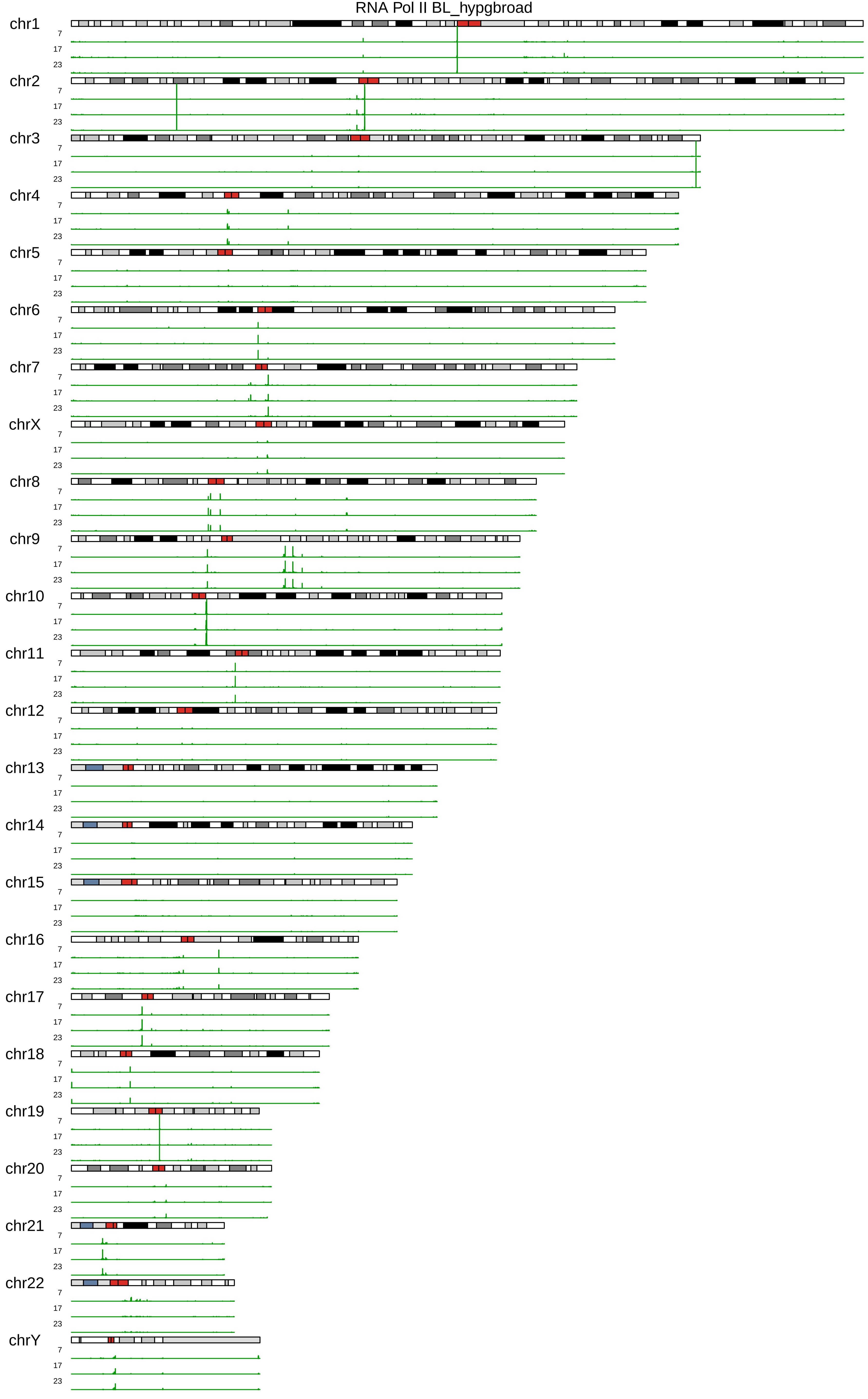

RNA Pol II BL_hypgbroad
chr1
chr2
chr3
chr4
chr5
chr6
chr7
chrX
chr8
chr9
chr10
chr11
chr12
chr13
chr14
chr15
chr16
chr17
chr18
chr19
chr20
chr21
chr22
chrY
7
17
23

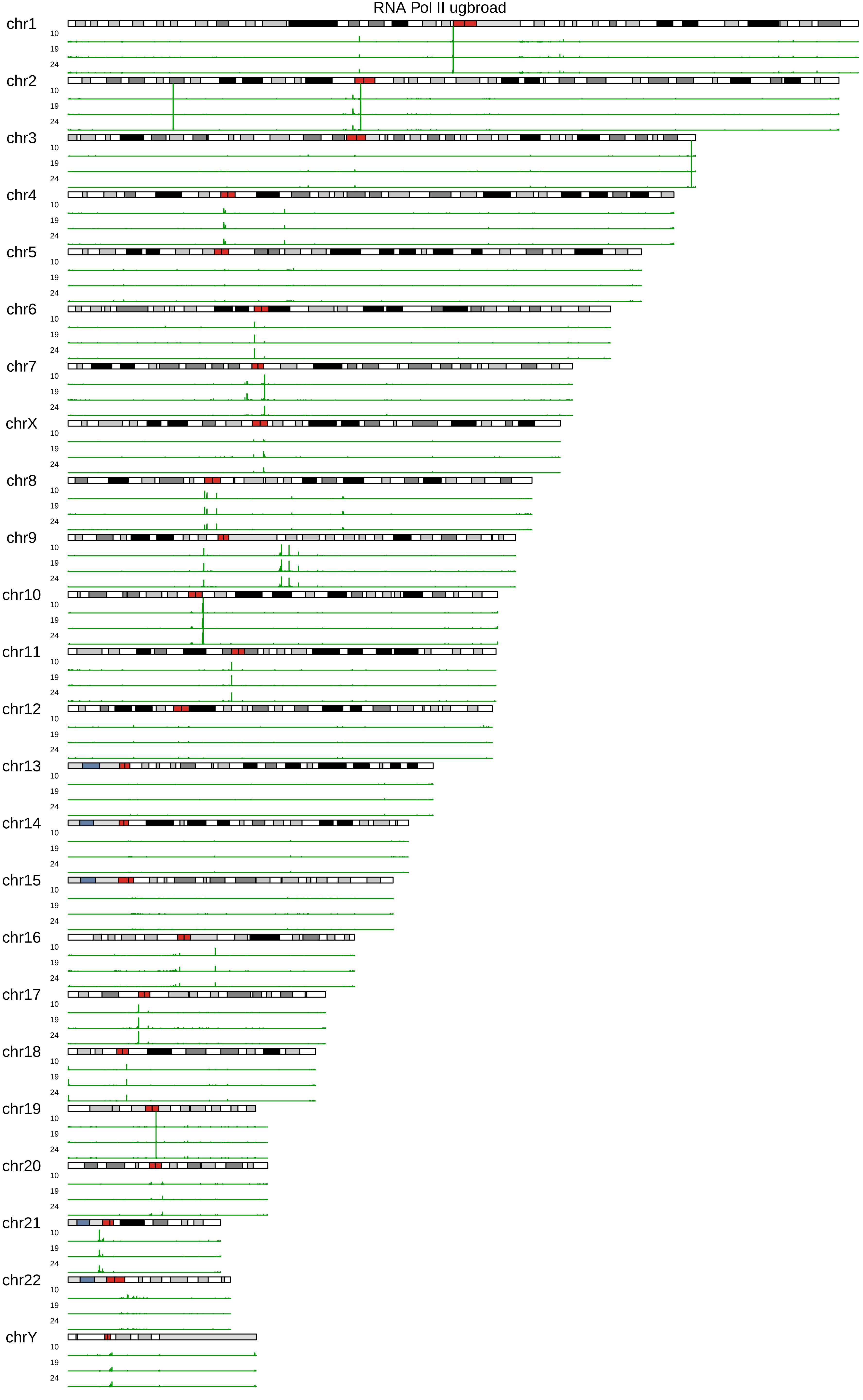
RNA Pol II ugbroad
chr1
chr2
chr3
chr4
chr5
chr6
chr7
chrX
chr8
chr9
chr10
chr11
chr12
chr13
chr14
chr15
chr16
chr17
chr18
chr19
chr20
chr21
chr22
chrY
10
19
24
