# Supplementary material for: Rapid Downregulation of H3K4me3 Binding to Immunoregulatory Genes in Altered Gravity in Primary Human M1 Macrophages
Source: Int J Mol Sci. 2022 Dec 29;24(1):603. doi: 10.3390/ijms24010603 (PMC9820304; doi:10.3390/ijms24010603)
Supplement: Supplementary file 1 [file ijms-24-00603-s001.zip › Figure S3 - Broad Peak Calling.pdf]

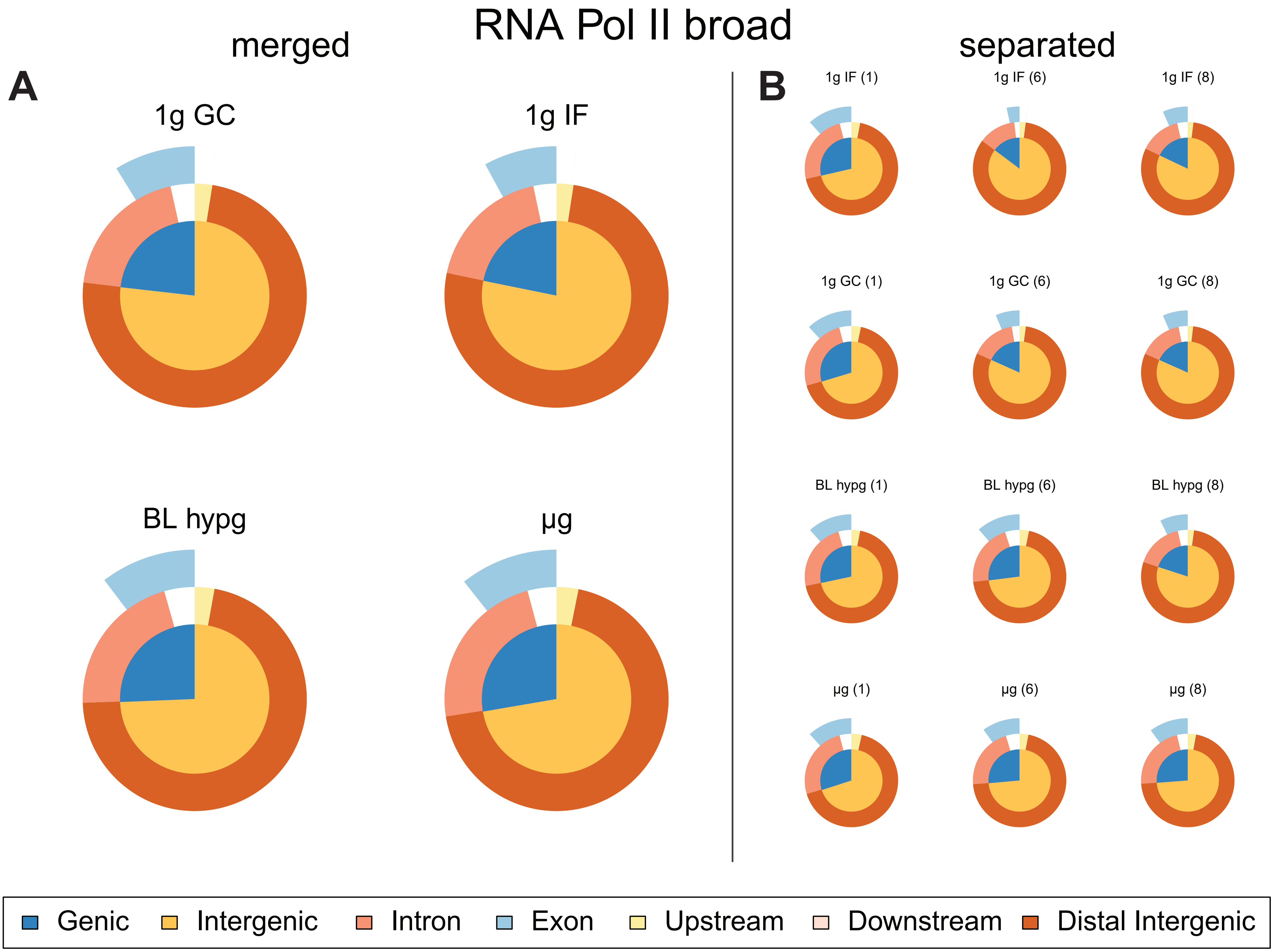
RNA Pol II broad
merged
separated
A
B
1g GC
1g IF
BL hypg
µg
1g IF (1)
1g IF (6)
1g IF (8)
1g GC (1)
1g GC (6)
1g GC (8)
BL hypg (1)
BL hypg (6)
BL hypg (8)
µg (1)
µg (6)
µg (8)
Genic
Intergenic
Intron
Exon
Upstream
Downstream
Distal Intergenic
