# Supplementary material for: Rapid Downregulation of H3K4me3 Binding to Immunoregulatory Genes in Altered Gravity in Primary Human M1 Macrophages
Source: Int J Mol Sci. 2022 Dec 29;24(1):603. doi: 10.3390/ijms24010603 (PMC9820304; doi:10.3390/ijms24010603)
Supplement: Supplementary file 1 [file ijms-24-00603-s001.zip › Supplementary Figures.pdf]

Supplementary Figures

Supplementary Figure S1: Raw RNA sequencing reads of the performed ChIP-seq experiment are displayed along the full human chromosome set. Genomic location is indicated by the mega-basepair scale on each chromosome. Sequencing reads are averaged along three biological replicates per gravity condition per antibody, originating from three different blood donors to account for the biological spread along different donors. In-hardware 1g ground control condition is in green, 1g in flight reference in blue, hypergravity phase sample in yellow and microgravity in red. The signals consist of enriched ChIP peaks, background noise and unspecific peaks that are not generated by antibody immunoprecipitation. Regions that colocalize with the ENCODE blacklist are excluded and are displayed as flat signal. A) Raw reads for histone antibody H3K4me3. B) Raw reads for RNAPol2 antibody.

Supplementary Figure S2: Peaks called by MOSAiCS in sharp peak mode for RNA Pol II and H3K4me3 and broad peak HMM mode for RNA Pol II are visualized along the human chromosome set. Each sample is shown separately but grouped by gravity condition. Peak height represents ChIP peak intensity. For better visualization, the 10 highest peaks have all been set to the peak height of the 10th strongest peak. The small numbers left of each line represent the internal sample numbers.

Supplementary Figure S3: Classification of peaks from the RNA Pol II antibody dataset, called by MOSAiCS in broad peak Hidden Markov Model mode. The inner circle represents the fraction of genic and intergenic sequences, the outer circle rings represent the fractions of intronic/exonic sequences for genic sequences and for peaks upstream/downstream and distant from transcription start sites. **(A)** Replicates merged by gravity condition. **(B)** Each replicate displayed separately. Blood donor number is indicated in brackets.

Supplementary Figure S4: To perform a stability analysis of the results versus peak caller method, the differential analysis was conducted with a different peak calling technique: Differential peak binding for RNA Pol 2 based on broad peak calling with a Hidden Markov Model approach by MOSAiCS. **(A)** Significantly differentially bound peaks are visualized along the human chromosome set, increased binding in comparison to 1g in blue, decreased binding in red. Line numbers indicate the different comparisons, explained in the legend box. **(B)** Characterization of binding locations as Venn pies for all three comparisons. Number of significantly altered peaks are indicated below.
