# Supplementary figures and images for: Rapid Downregulation of H3K4me3 Binding to Immunoregulatory Genes in Altered Gravity in Primary Human M1 Macrophages
Source: Int J Mol Sci. 2022 Dec 29;24(1):603. doi: 10.3390/ijms24010603 (PMC9820304; doi:10.3390/ijms24010603)

**A****H3K4me3**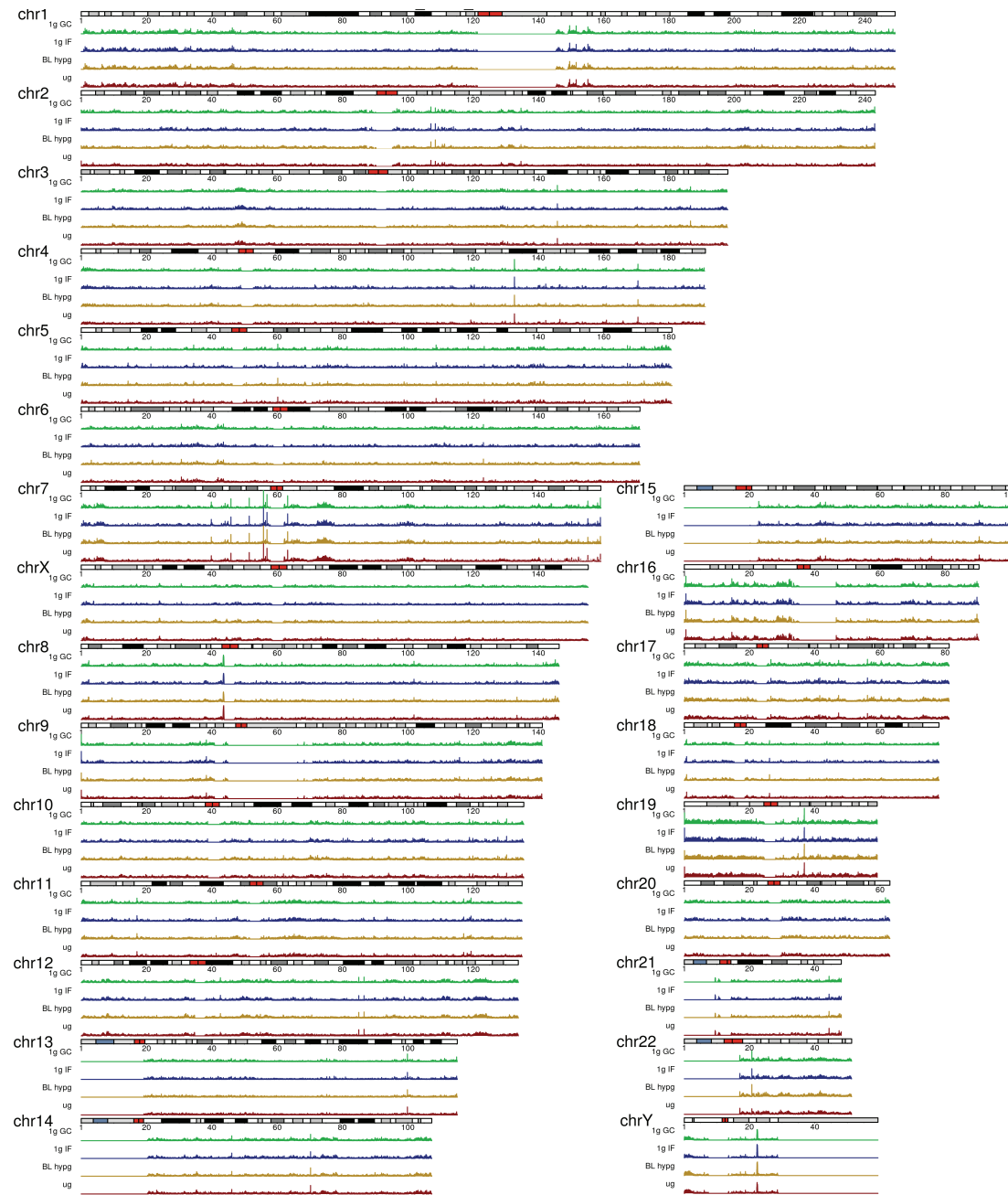**B****RNAPol2**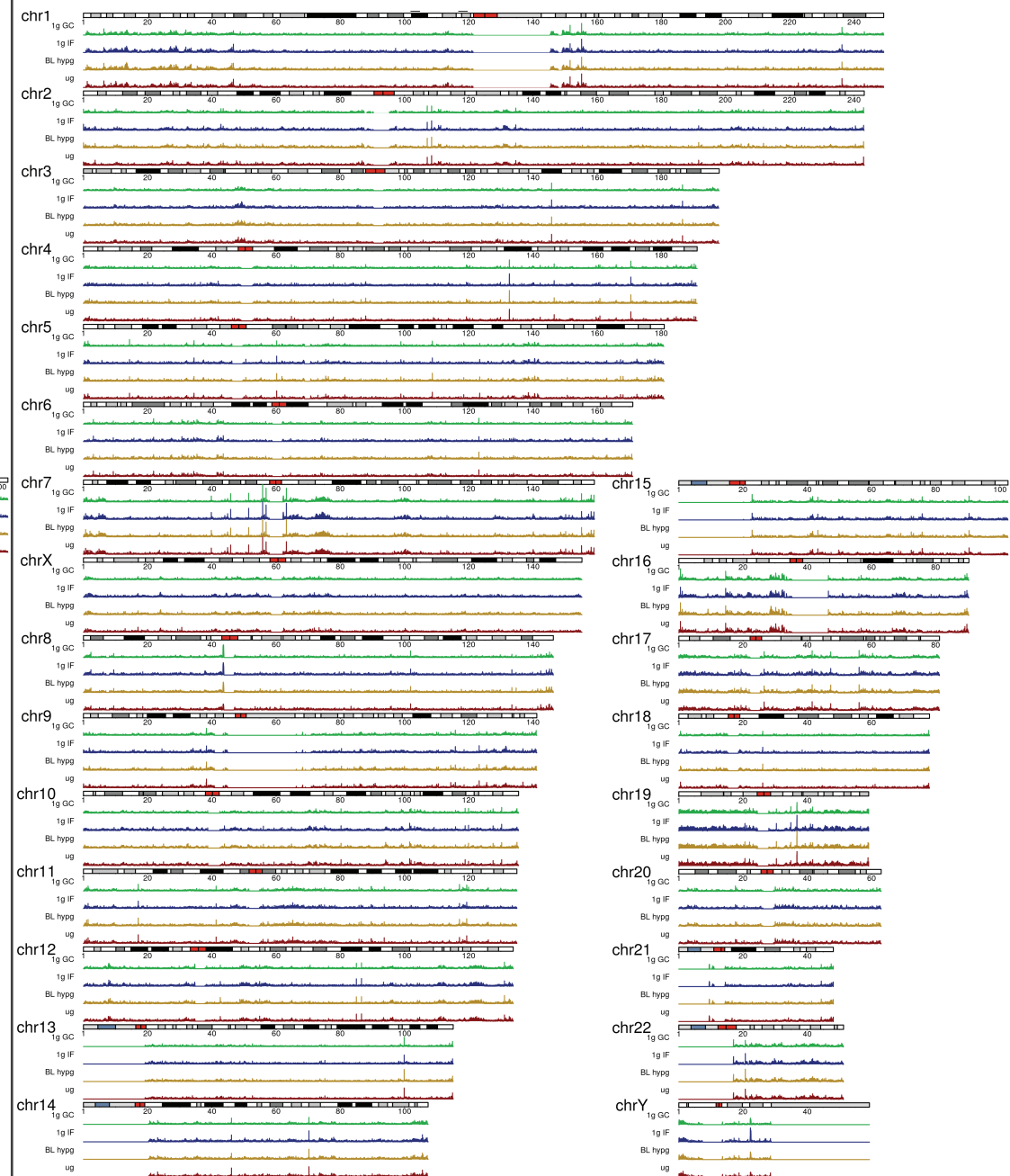

Supplement: Supplementary file 1 [file ijms-24-00603-s001.zip › Figure S1 - Raw Reads.pdf]

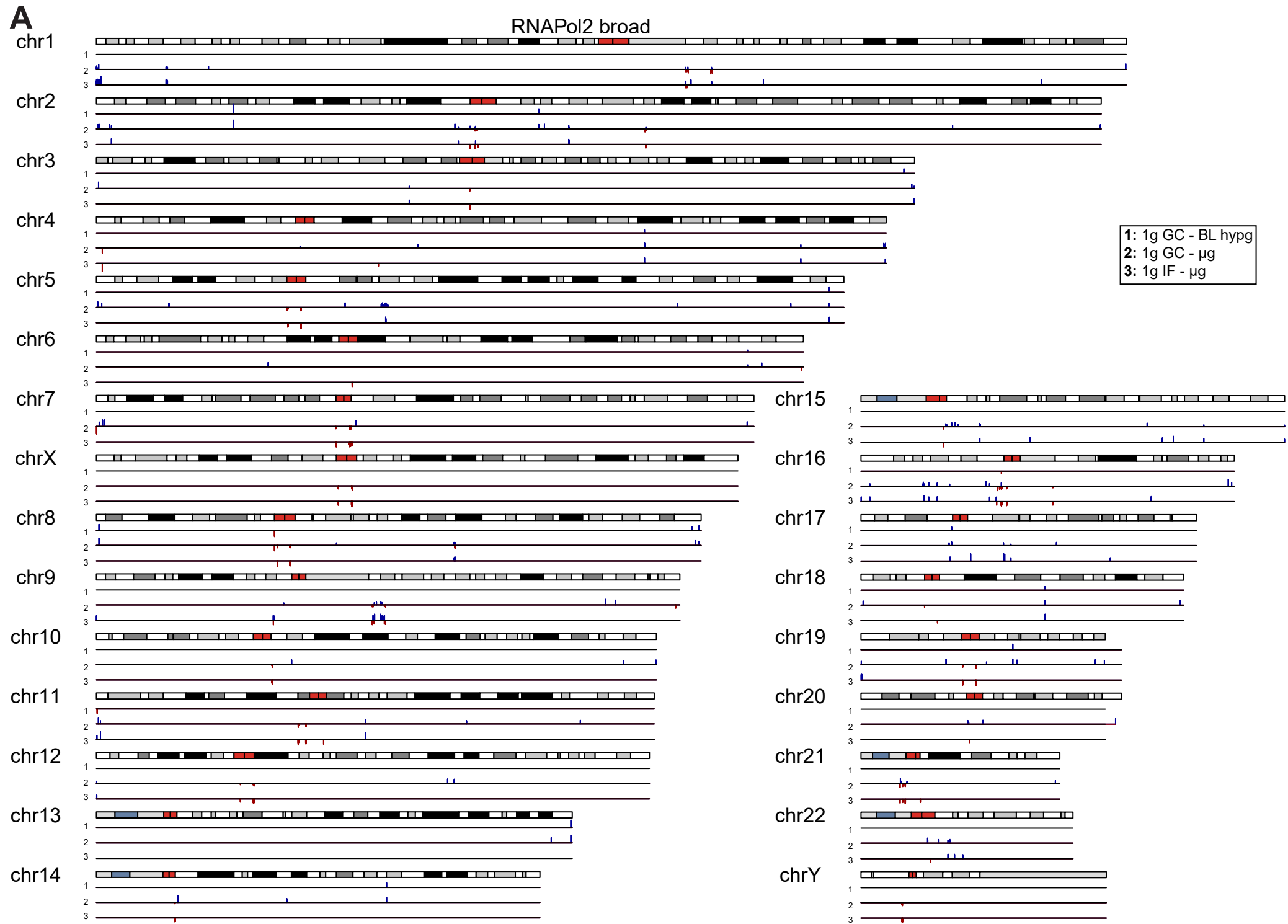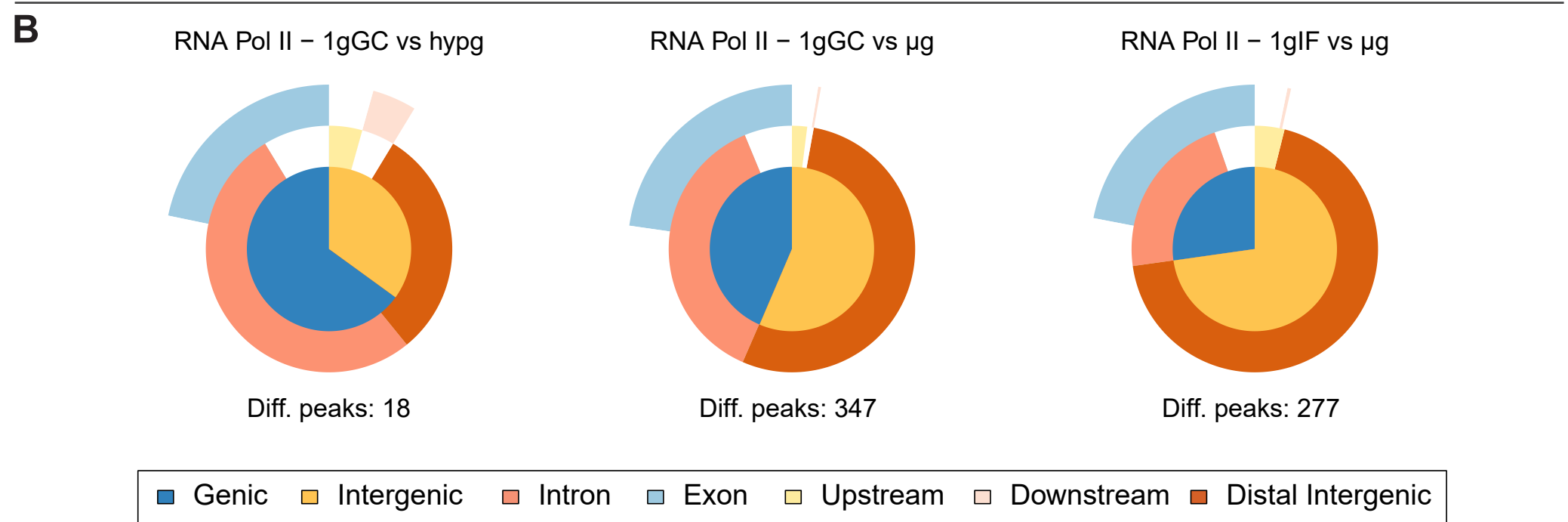

Supplement: Supplementary file 1 [file ijms-24-00603-s001.zip › Figure S4 - Broad Calling_differential.pdf]
